# Solvent Stabilization of Protic Oxonium/Ammonium Intermediates in Cation Radical Cyclization Reactions Investigated via Computational Approaches

**DOI:** 10.1021/acs.joc.6c00258

**Published:** 2026-07-07

**Authors:** Shahriar N. Khan, John H. Hymel, Jesse G. McDaniel

**Affiliations:** † School of Chemistry and Biochemistry, 6146 Georgia Institute of Technology, Atlanta, Georgia 30332, United States; ‡ Oak Ridge Leadership Computational Facility, 1372 Oak Ridge National Laboratory, Oak Ridge, Tennessee 37830, United States

## Abstract

Anodic intramolecular cyclization reactions have substantial synthetic utility for formation of cyclic carbon–carbon or carbon–heteroatom bonds. For cases of intramolecular trapping of a cation radical by a protic nucleophile, the cyclization step coincides with a substantial increase in substrate acidity and thus may exhibit particularly pronounced solvent effects. In this computational work, we employ both quantum chemical (QM) and quantum mechanics/molecular mechanics (QM/MM) methods to compute solvent effects on free energy profiles for cyclization and deprotonation reaction steps for cation radical intermediates of substrates representative for anodic intramolecular cyclizations. We find substantial solvent contribution to the thermodynamic driving force for cation radical cyclization; for example, methanol and tetrahydrofuran solvents provide ∼30–35 kJ/mol driving force to form cyclic oxonium cation radicals and ∼15–25 kJ/mol driving force to form cyclic ammonium cation radicals, compared to baseline reactions in dichloromethane solvent. Given that these solvent shifts are on par with the innate cyclization reaction thermodynamics, the choice of solvent plays a crucial role in promoting/driving the cation radical cyclization step. Methanol is particularly effective at facilitating rapid deprotonation of the cyclic cation radical intermediate, which may lead to the full electrochemical process (e.g., second electron transfer) proceeding heterogeneously at the anode.

## Graphical abstract

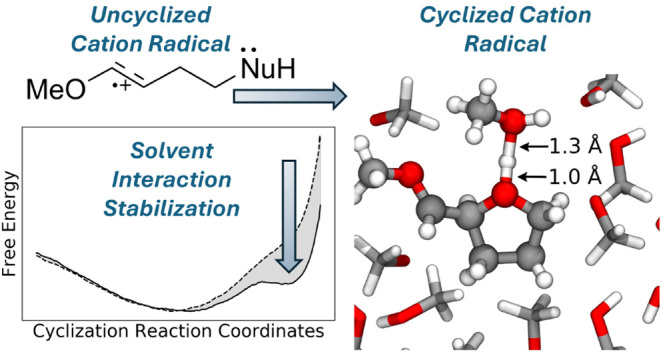

## 1 Introduction

Cation radical-initiated cyclizations are key steps in various electrochemical, photoredox, or chemical oxidation-based synthetic transformations. While the overall transformations differ—for example, anodic electrolysis reactions typically proceed via a net two-electron oxidation, while photochemical pathways are net redox neutral—the initial mechanistic steps are similar, involving intramolecular trapping of a cation radical group by a nucleophile. Thus, the stability/reactivity of the cyclized cation radical intermediate is a common, important factor for product selectivity/yield among the different synthetic routes. When protic nucleophiles are involved, the cyclization step results in a dramatic increase in acidity of the cation radical intermediate; for example, trapping with amine or alcohol nucleophiles results in acidic oxonium or ammonium groups. In these cases, the driving force for the cation radical cyclization is intimately connected with the stability of the resulting acidic moiety and thus may be substantially altered by the nature of the solvent and/or the presence of a base. Because in some cases, these reactions occur within complex environments, e.g., within the electrical double layer near an electrode surface for electrolysis processes, it is important to develop a mechanistic understanding of solvent effects on the key cation radical cyclization step.

Extensive work by the Moeller group^1–19^ and others^20–26^ on anodic intramolecular coupling reactions has provided substantial mechanistic insight into cation radical cyclizations. Electrochemical transformations often employ substrates with electron-rich olefins such as enol ethers, vinyl sulfides, or ketene dithioacetal groups as electroauxiliaries, which, when strategically paired with appropriate nucleophiles and subject to optimal reaction conditions, result in cyclic carbon–carbon or carbon–heteroatom bond formation via cation radical intermediates.^1–4,9–11,16,18,19^ The success and product yield of such anodic cyclization reactions depend on both the chemical nature of the cation radical and the nucleophilic trapping group, as well as external reaction conditions.^2–4,6,7^ Moeller and co-workers have generally observed that cyclization reactions forming carbon–heteroatom bonds work best with less polar cation radicals, while cyclization with carbon–carbon bond formation is optimal with more polar cation radicals.^1,2,7,10^ However, because in all cases, the cation radical cyclization is thought/shown to be reversible, the kinetics of the subsequent reaction steps can substantially alter the reaction yield.^2–4,6,7^ This is because the cation radical is generally susceptible to a variety of competitive side reactions, including solvent nucleophilic attack, and possibly deprotonation/decomposition, and the cyclic structure is only “locked-in” after a second oxidation step.^2^

In this work, we focus on anodic cyclization reactions of substrates with either enol ether or ketene dithioacetal cation radicals that are trapped by either alcohol, amine, or sulfonamide nucleophiles.^1,2,4,6,10,11,16,27^ We consider electrochemical transformations that proceed via a net two-electron oxidation, although much of the resulting discussion has relevance to cation radical cyclizations within photochemical or chemical oxidation reactions. A schematic of the electrochemical oxidative coupling reactions is shown in Figure 1. Following oxidation at the anode, intramolecular nucleophilic attack forms the cyclic cation radical intermediate. This cyclization step is typically reversible.^2–4,6,7^ Subsequent deprotonation and loss of a second electron “lock in” the cyclic structure; in general, the order of these microscopic steps could vary. The final step is trapping of the cation by the solvent, such as methanol, and a second deprotonation to form the neutral, cyclic product. Depending on the magnitudes of microscopic rate constants, the second oxidation shown in Figure 1 can generally occur either heterogeneously at the anode surface or homogeneously via a second-order disproportionation process, in either case typically with a large thermodynamic driving force.^28^

**Figure 1:**
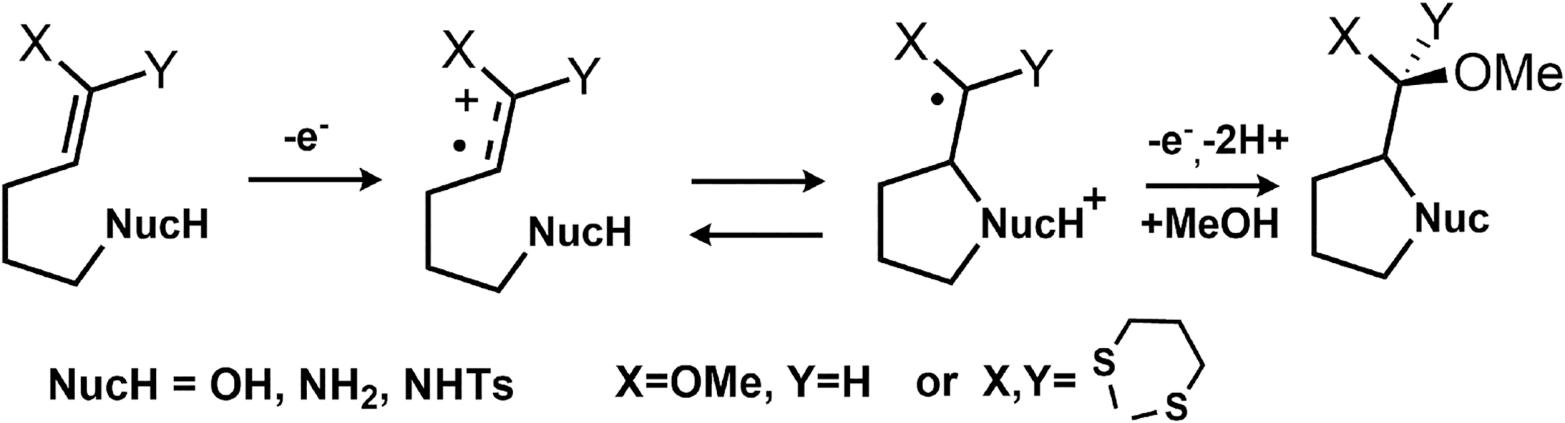
Schematic of the oxidative intramolecular coupling reaction for substrates containing enol ether or ketene dithioacetal electroauxiliary groups with alcohol, amine, or sulfonamide nucleophiles.

Several mechanistic assumptions implicit in the reaction schematic in Figure 1 will be subject to computational investigation. For example, it is assumed that the cyclization step involves the protic nucleophile rather than the deprotonated form (e.g., −OH vs −O^–^), as consistent with the experimental conditions of neutral pH without addition of a base; experiments conducted under basic conditions would likely proceed via different elementary mechanisms.^4^ In this case, the cyclization step involves the formation of a highly acidic cyclized cation radical intermediate with expected major solvation contribution to the driving force. Second, because the cation radical cyclization is reversible (Figure 1), the order and kinetics of the subsequent elementary deprotonation and electron transfer steps are important considerations that may affect reaction yield.^2–4,6,7^ In this regard, the deprotonation kinetics is likely to vary with the type of solvent. If the deprotonation is relatively slow and depending on the electronic structure of the cyclized cation radical, it is possible that the second oxidation/electron transfer could occur before deprotonation, but this would depend on the substrate, as will be investigated. A final consideration is the compatibility of the nucleophile (−OH, –NHR, –NHTs) and the electroauxiliary group (enol ether or ketene dithioacetal) for the hypothesized reaction mechanism. Particularly for the amine nucleophiles, their relatively low oxidation potentials may entail that oxidation produces a nitrogen radical rather than the schematic electronic structure in Figure 1, which would result in a different mechanistic pathway.

In this work, we employ both quantum chemical (QM) and quantum mechanics/molecular mechanics (QM/MM) methods to compute both energetics and free energy barriers of the various elementary steps involved in cation radical cyclization reactions of the type shown in Figure 1. The scope of substrates investigated is motivated by previous studies by the Moeller group, demonstrating both mechanisms and practical utility of such anodic cyclization reactions.^1,2,4,6,11,16^ We utilize density functional theory (DFT) to compute oxidation potentials for both the unreacted substrate and the final cyclization product for ten substrates with different combinations of the nucleophile (−OH, –NHR, –NHTs) and the electroauxiliary group (enol ether or ketene dithioacetal). We compute the electronic structure/spin density of the cation radical intermediates to explore the radical localization on the functional groups and its consistency with the proposed cyclization mechanism. From this screening, we downselect to four substrates with alcohol and sulfonamide nucleophiles and enol ether or ketene dithioacetal electroauxiliary groups. For the latter four substrates, we utilize QM/MM free energy simulations to compute cyclization reaction free energies of the cation radical intermediate in four different solvents: methanol, tetrahydrofuran, acetonitrile, and dichloromethane. From these computations, we highlight the role of the solvent in driving the cyclization reaction by stabilizing and complexing with the acidic proton of the cation radical heterocycle. For one of the representative substrates, we further compute free energy profiles for deprotonation of the cyclic cation radical intermediate to elucidate differences between the solvents in the rate of the subsequent deprotonation step. The overarching conclusion is that the solvent plays a significant role in stabilizing the key cyclized cation radical intermediate via complexing with the acidic proton. Methanol, tetrahydrofuran, and acetonitrile all provide substantial driving force for the cyclization process as compared to the dichloromethane solvent, with dichloromethane not effectively complexing/stabilizing the acidic proton of the cation radical heterocycle. Methanol is unique among the solvents in that it facilitates a low-barrier and favorable deprotonation step to the cyclic radical, which can then undergo further oxidation.

## 2 Methods

### 2.1 Quantum Chemical Calculations

Quantum chemical calculations were performed with ORCA v6.0 software.^29,30^ Calculations were conducted at the PBE0-D3/6-31G* level of theory, utilizing the CPCM­(methanol) implicit solvent model and unrestricted Kohn–Sham (UKS) for doublet spin systems (cation radicals). The ‘TightSCF’ convergence threshold was utilized, and energetics are reported following geometry optimization to the relevant stationary point (reactant/product) on the potential energy surface. Redox potentials were further computed at the RI-MP2/6-31G*/CPCM­(methanol) level of theory for comparison, based on geometries optimized at the DFT level.

The PBE0-D3/6-31G* level of theory is utilized consistently throughout this work for QM/MM-MD simulations, as will be described subsequently. For the investigated solution-phase cation radical reactions, there is currently “no perfect choice” of computational approach to accurately predict both the substantial electronic and solvation contributions to reaction free energies.^31^ It is well-known that self-interaction error intrinsic to DFT functionals manifests in quantitative error in relative energetics of organic cation radical intermediates;^32–36^ based on MP2/cc-pVTZ benchmarks in Table S3, we benchmark such errors to be ∼5–20 kJ/mol for a subset of substrates investigated in this work. This error would be larger if not for error cancellation (i.e., small basis set, self-interaction error), as demonstrated by benchmarks with larger basis sets (e.g., PBE0-D3/6-311G*). We have also demonstrated the effect of deploying a polarization function on the H atoms (6-31G** basis sets) in Figure S1, comparing the H-bond distances of the complexes for 6-31G* and 6-31G** basis sets. The primary motivation for the choice of level of theory (PBE0-D3/6-31G*) is to facilitate QM/MM explicit solvent free energy predictions, which would be computationally intractable with higher levels of electronic structure theory. For reactions of ions or neutral zwitterionic species, implicit solvent models may exhibit errors of 20 kJ/mol or more in reaction free energy predictions,^31,37^ while the QM/MM explicit solvent simulations utilized in this work are expected to provide an accurate description of such solvation energetics.

### 2.2 Quantum Mechanics/Molecular Mechanics Simulations

DFT-based, QM/MM molecular dynamics (QM/MM-MD) simulations in tandem with free energy sampling were utilized to compute cyclization and deprotonation reactions of the cation radical substrates in various solvents. The systems consisted of a simulation box of 2000 solvent molecules with the solvated organic substrate/intermediate, modeled with periodic boundary conditions. Four different solvents were studied, namely, methanol (MeOH), tetrahydrofuran (THF), acetonitrile (ACN), and dichloromethane (DCM), and the initial simulation system was constructed utilizing PackMol software,^38^ followed by density equilibration for 50 ns in the NPT ensemble at 300 K and 1 bar. System equilibration was done with classical molecular dynamics (MD) utilizing the OPLS-AA force field^39^ to model both the solvent molecules and organic substrates. The force field parameters for the cation radical substrates are taken to be the same as their neutral counterparts (i.e., default OPLS-AA parameters), except for the charges. For the cation radical substrates, partial atomic charges were parametrized based on DFT calculations at the PBE0-D3/6-31G* level of theory, followed by distributed multipole analysis (DMA)^40^ and subsequent atomistic charge fitting;^41,42^ the parametrized charges are given in Table S1 of the Supporting Information. The classical MD simulations (for equilibration) were performed with OpenMM v7.7 software^43^ and utilized a time step of 1 fs, with a Langevin thermostat and a Monte Carlo barostat with frequencies of 1 and 0.1 ps^–1^, respectively. Particle mesh Ewald (PME)^44^ was utilized for long-range electrostatic interactions, and a cutoff distance for van der Waals interactions was set at 1.4 nm.

QM/MM-MD simulations were then conducted starting from the equilibrated systems from classical MD. The MM substrate geometry was then replaced with the optimized structure at the PBE0-D3/6-31G* level of theory, followed by subsequent equilibration of the full system with QM/MM-MD for 2 ps of simulation, preceding production simulations. The QM/MM simulations were conducted utilizing PyDFT-QMMM software,^45^ as interfaced with the Psi4 quantum chemistry package (v1.5)^46^ and the OpenMM molecular dynamics package (v7.7).^43^ The QM/MM Hamiltonian is of the typical form,^47–52^ utilizing electrostatic embedding of MM point charges within the Kohn–Sham Hamiltonian and Lennard–Jones (LJ) interactions between QM and MM regions. The electrostatic embedding and LJ interactions between QM and MM regions were evaluated based on a group-based, centroid cutoff of 1.4 nm, as evaluated at each time step. The QM region is modeled at the PBE0-D3/6-31G* level of theory with a 50/194 Lebedev–Treutler quadrature grid, and the MM region is modeled with the OPLS-AA force field.^39^ For all reactions studied, the QM region consisted of the cation radical substrate and additionally either zero, one, or two neighboring solvent molecules (as will be discussed in context), with the remainder of the solvent belonging to the MM region. The QM/MM-MD simulations were conducted in the NVT ensemble, utilizing a time step of 1 fs with a Langevin thermostat set at a temperature of 300 K and a friction coefficient of 5 ps^–1^. At each time step, the self-consistent field (SCF) optimization of the Kohn–Sham, QM/MM Hamiltonian utilized as initial guess the converged wave function of the previous time step; unrestricted Kohn–Sham (UKS) was used to treat the doublet spin systems (cation radicals).

We note that such QM/MM simulations that include solvent molecules in the QM region often employ “adaptive” (or “constrained”) approaches to facilitate (or prevent) solvent exchange between QM and MM regions.^53,54^ We do not utilize/consider such approaches here, as the QM solvent molecules are intrinsically “localized” in close contact with the cation radical substrates during the reaction, due to strong energetic “complexation” with the acidic proton of the substrate. Analysis and discussion of this effect is given in Section S4 of the Supporting Information (Figure S1). The only exception is for noncomplexing dichloromethane solvent, in which some solvent exchange (QM solvent exchange with MM solvent for close contact) is observed due to weak interactions with the substrate. However, we view this as a minor issue, as the computed reaction free energies are insensitive to solvent representation in this particular case.

### 2.3 Free Energy Simulations

Both umbrella sampling and metadynamics techniques were utilized in tandem with QM/MM-MD to study different cation radical reactions; umbrella sampling was utilized to study cyclization reactions, while metadynamics was utilized to study subsequent deprotonation reactions. In all cases, the PLUMED2 software package (v2.8.0)^55^ (as interfaced as a plugin to PyDFT-QMMM^45^) was utilized for free energy sampling simulations. Umbrella sampling for the cation radical cyclization reactions utilized biasing umbrellas with force constants of 1200 kJ/mol/Å^2^, with umbrellas evenly spaced along the cyclization reaction coordinate at intervals of 0.1 Å. The variables used to define the reaction free energy profiles are the C–O or C–N distances of the formed bond during cyclization for alcohol or sulfonamide nucleophiles; this distance coordinate spans 1.3 to 3.5 Å with a spacing of 0.1 Å for the umbrella sampling. The starting configuration for each umbrella simulation was generated from a 2 ps steering trajectory that “pulled” the molecular configuration to the target umbrella bias coordinate. Subsequently, a 25 ps production trajectory of QM/MM-MD was conducted at each umbrella position, corresponding to 575 ps of total simulation to construct the full reaction free energy profile. The Weighted Histogram Analysis Method (WHAM) was used to reverse-bias the distribution to generate the corresponding free energy surface.

To investigate subsequent cation radical deprotonation reactions, two-dimensional (2D) free energy profiles were computed utilizing well-tempered metadynamics in combination with QM/MM-MD.^56^ Metadynamics simulations were conducted utilizing the multiple-walker approach in PLUMED2,^55^ enabling parallelization across multiple simultaneous trajectories/walkers. We used 32 walkers with a bias factor of 100 kJ/mol. Of these walkers, 16 were initialized from configurations of the cation radical species prior to cyclization and deprotonation, while the remaining 16 began from cyclized configurations with the excess proton located on the nearest solvent molecule. In simulations with multiple solvent molecules within the QM region, the latter 16 walkers started with the acidic proton shared/positioned equally between two QM solvent molecules. Gaussian biases with an initial height of 2 kJ/mol and widths of 0.1 Å (for both CVs) were deposited every 100 fs. Simulations with a single quantum mechanically (QM) treated solvent molecule ran for 40 ps per walker, whereas simulations incorporating two QM solvent molecules were run for 25 ps per walker.

Two collective variables were utilized for the well-tempered metadynamics simulations: the five-membered ring cyclization coordinate (identical to umbrella sampling simulations described above), and a proton transfer coordinate^57^ (*R*_PT_), defined as

1 $$R_{\mathrm{PT}}=R\left(O_{\mathrm{Alc}}-H^{+}\right)-R\left(H^{+}-A_{\mathrm{solv}}\right)$$

where *R*(*O*_Alc_–*H* ^+^) is the distance between the alcohol oxygen atom of the substrate and the transferring proton, and *R*(*H* ^+^–*A*_solv_) is the distance between the transferring proton and the proton-accepting atom of the QM solvent molecule, which is oxygen for methanol and THF and nitrogen for acetonitrile (note that these simulations were only conducted for substrates with alcohol nucleophiles). Consequently, negative *R*_PT_ values correspond to the proton residing on the cation radical substrate, while positive values indicate proton transfer to the QM solvent. In simulations with multiple QM solvent molecules, a smooth minimum function selected the shortest *R*(*H* ^+^–*A*_solv_) distance at each simulation snapshot. To restrict exploration of the simulation to the region of interest, half-harmonic restraining potentials (walls) were applied. For the cyclization coordinate, an upper wall at 3.0 Å with a force constant of 1000 kJ/mol was imposed. For *R*_PT_, lower and upper walls were placed at −2.0 and 2.0 Å, respectively, each with a force constant of 1000 kJ/mol. Additionally, a lower wall with a force constant of 1000 kJ/mol was positioned at 2.0 Å along the carbon–oxygen distance corresponding to the formation of the undesired six-membered ring product, preventing side reactions.

## 3 Results and Discussion

The substrates considered for potential anodic intramolecular coupling reactions are shown in Figure 2. These substrates contain either enol ether or ketene dithioacetal electroauxiliary groups, with either alcohol, amine, or sulfonamide nucleophiles, and are chosen to be similar to substrates that have been experimentally investigated by Moeller and co-workers.^1,2,4,6,10,11,16^ It is important to note that not all substrates in Figure 2 may be good candidates for practical anodic intramolecular coupling reactions if the products are not oxidatively stable at the anodic working potential. Furthermore, whether the radical character of the oxidized substrate is localized on the electroauxiliary group (rather than the nucleophile), as in the mechanism shown in Figure 1, may depend on the specific combination of the electroauxiliary/nucleophile.

**Figure 2:**
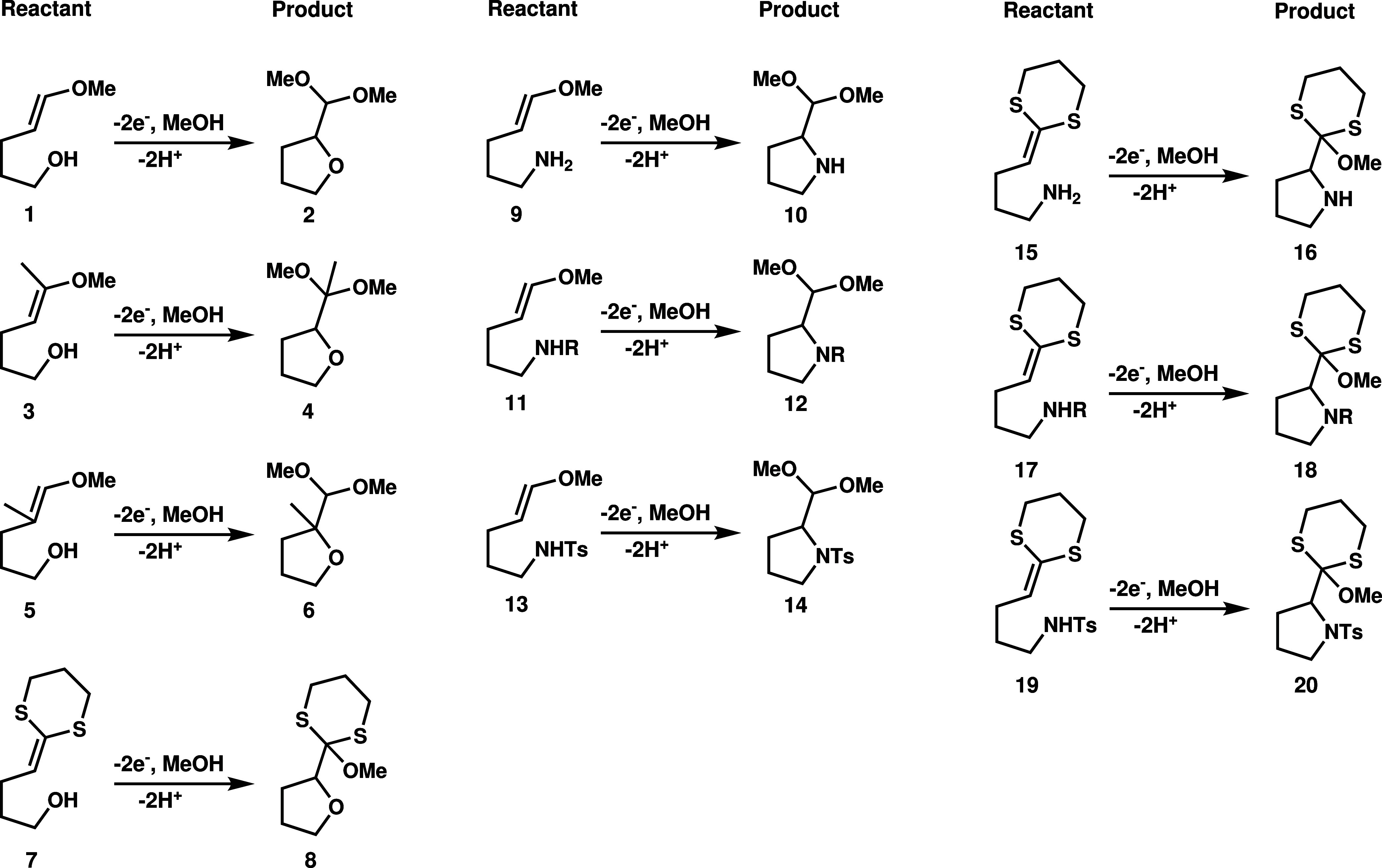
Substrates considered for potential anodic intramolecular coupling reactions. Substrates contain either enol ether or ketene dithioacetal electroauxiliary groups, with either alcohol, amine, or sulfonamide nucleophiles (“*R*” represents methyl substituents).

In Section 3.1, we compute and discuss oxidation potentials of both the initial substrate and target product to determine which substrates in Figure 2 are potentially practical for oxidative coupling reactions. We further analyze the electronic structure (e.g., radical spin density) of the oxidized intermediate to investigate the localization of radical/cation character and whether the proposed cation radical coupling pathway in Figure 1 would be relevant. Based on these findings, we then downselect to four representative substrates and explore the intramolecular cyclization reactions of the corresponding cation radical intermediates. This is discussed in Section 3.2, in which we present reaction free energy profiles computed with QM/MM simulations in methanol, tetrahydrofuran, acetonitrile, and dichloromethane solvents, to explore the solvent-modulated reactivity of the cation radical intermediate. Finally, in Section 3.3, we present and discuss QM/MM simulations for deprotonation of the cyclic cation radical intermediates within the different solvents that provide insight into the (in)­stability of these highly acidic cation radical intermediates.

### 3.1 Oxidation Potentials of the Substrates and Products and the Electronic Structure of Oxidized Cation Radicals

The oxidation potentials for the substrates shown in Figure 2 depend on whether the electroauxiliary group is an enol ether or ketene dithioacetal and the type of nucleophilic group (alcohol, amine, or sulfonamide). For comparison, oxidation potentials of unfunctionalized substrates with enol ether or ketene dithioacetal electroauxiliaries are ∼1.40 and ∼1.06 V vs Ag/AgCl, respectively, as measured by cyclic voltammetry.^1,11,19^ However, the presence of the additional nucleophilic group tends to lower the oxidation potential by ∼0.1–0.4 V relative to the bare electroauxiliary group, for rapid cyclization reactions.^1,3,5,9,11^ This effect has been previously rationalized as due to a “Nernstian shift” that occurs when the subsequent chemical step following oxidation is very rapid.^1,3,5,11,58^ Another important point is that oxidation potentials of amines are ∼1.1–1.2 V vs Ag/AgCl,^11^ being relatively low compared to other (neutral) nucleophiles and pertaining to substrates **9, 11, 15**, and **17**. For such substrates, the oxidative stability of the target cyclization product is a central consideration/concern for the practicality of the intended anodic coupling reaction.

Utilizing density functional theory (DFT), we computed the oxidation potentials of substrates and their target products in Figure 2. Details of the calculations are given in Section 2; briefly, calculations were done at the PBE0-D3/6-31G* level of theory utilizing the CPCM­(methanol) implicit solvent model. This choice of theory was based on consistency with subsequent QM/MM simulations (Sections 3.2–3.3), with accuracy considerations discussed in Section 2. Additionally, redox potentials were computed at the RI-MP2/6-31G* level as benchmarks, utilizing the DFT-optimized structures. The oxidation potential relative to the standard hydrogen electrode (SHE) reference is computed as^59^

2 $$E_{\mathrm{rel},\mathrm{SHE}}^{{}^{\circ}}=\frac{\Delta_{r}G^{{}^{\circ}}\left(R|O\right)}{n_{e}F}-E{{}^{\circ}}_{\mathrm{abs}}\left(\mathrm{SHE}\right)$$

where Δ_r_ *G*°(*R*|*O*) is the free energy change for the oxidation of the reduced “*R*” (neutral substrate) to oxidized “*O*” (cation radical intermediate) substrate in solution, “*F*” is Faraday’s constant, and *n*_e_ = 1 is the number of electrons in the redox process. The value of *E*_abs_^°^(SHE) (4.44 V) is used for the absolute standard potential of SHE, to convert to the reference SHE scale.^59,60^ In our computations, we approximate Δ_r_ *G*°(*R*|*O*) as the energy difference between a geometry-optimized oxidized substrate and a geometry-optimized neutral substrate; we thus neglect thermal and zero-point vibrational contributions, as the purpose is primarily a qualitative comparison of trends across the different substrates.

Figure 3 provides the computed oxidation potentials for the substrates (both reactants and products) shown in Figure 2. The primary redox potential values in Figure 3 were computed at PBE0-D3/6-31G*/CPCM­(methanol), while values computed at the MP2 level of theory are given for reference in Table S2 of the Supporting Information. In general, oxidation potentials computed at the PBE0-D3/6-31G*/CPCM level are ∼0.3–0.5 V lower than those computed at the MP2/cc-pVTZ/CPCM level of theory (Table S2). The computed values in Figure 3 may be compared to experimentally determined oxidation potentials of similar substrates. Oxidation potentials for substrates similar to **7** and **15** have been reported as 0.95 and 0.60 V vs Ag/AgCl, respectively, by Xu and Moeller.^11^ The oxidation potential of a substrate similar to **1** and **3** (i.e., containing the enol ether electroauxiliary/alcohol nucleophile) was reported as 1.1 V vs Ag/AgCl by Redden and Moeller.^9^ These comparisons suggest decent agreement between our computed oxidation potentials and available experimental reference values. We note that a complication in the oxidation potential predictions is that the (oxidized) cation radical substrates typically exhibit multiple local energy minima structures; while the neutral substrate is stable in an elongated or “unfolded” structure, the cation radical can have “unfolded”, “folded”, and “cyclized” structures, which are all local energy minima.^61,62^ The oxidation potentials in Figure 3 are computed from “unfolded” or elongated minimum energy structures for both neutral and oxidized states (geometries are shown in Figure 4), which may provide an overestimate of oxidation potentials (and why DFT predictions appear closer to experiment than MP2/cc-pVTZ benchmarks in Table S2, via error cancellation).

**Figure 3:**
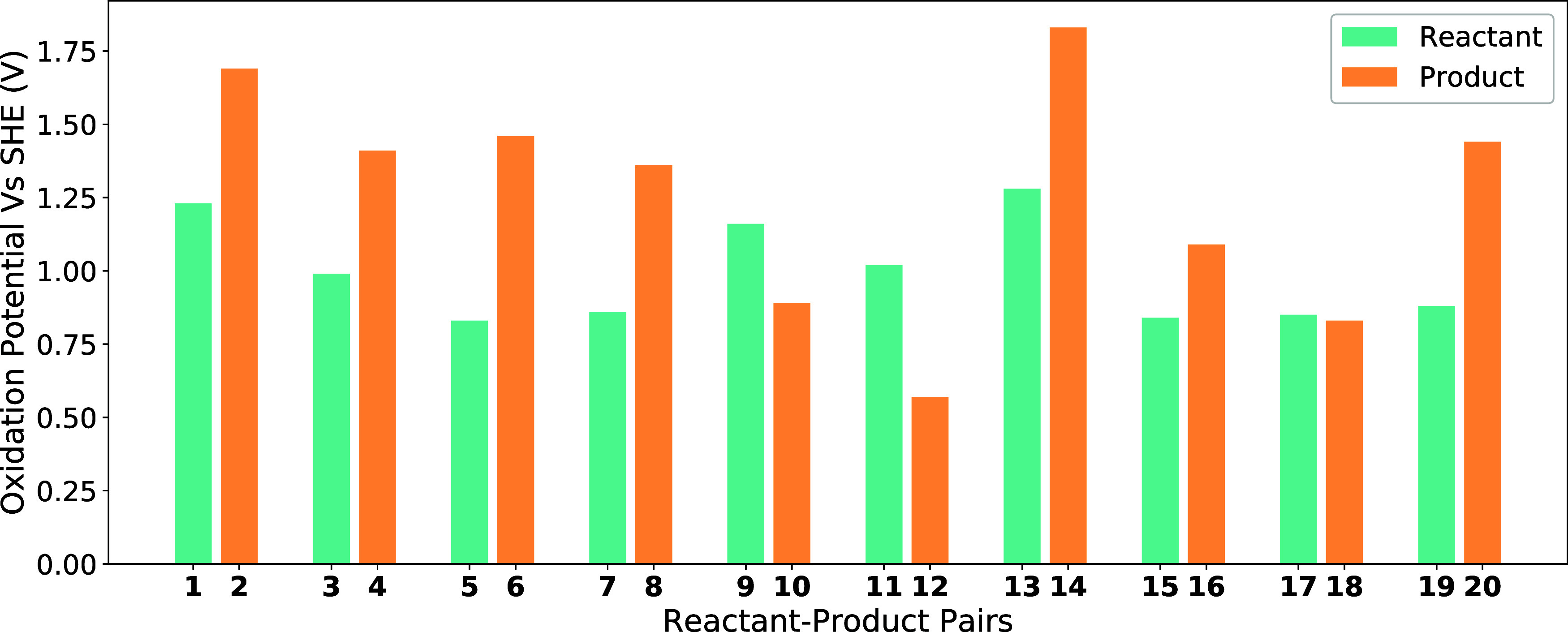
Oxidation potentials *E*_rel,SHE_^°^ (V) of substrates and products depicted in Figure 2 as computed at the PBE0-D3/6-31G*/CPCM­(methanol) level of theory as reported relative to the SHE reference. The corresponding oxidation potentials of the species computed at the RI-MP2/6-31G*/CPCM­(methanol) and RI-MP2/cc-pVTZ/CPCM­(methanol) levels on the DFT-optimized geometries are presented in Table S2.

**Figure 4:**
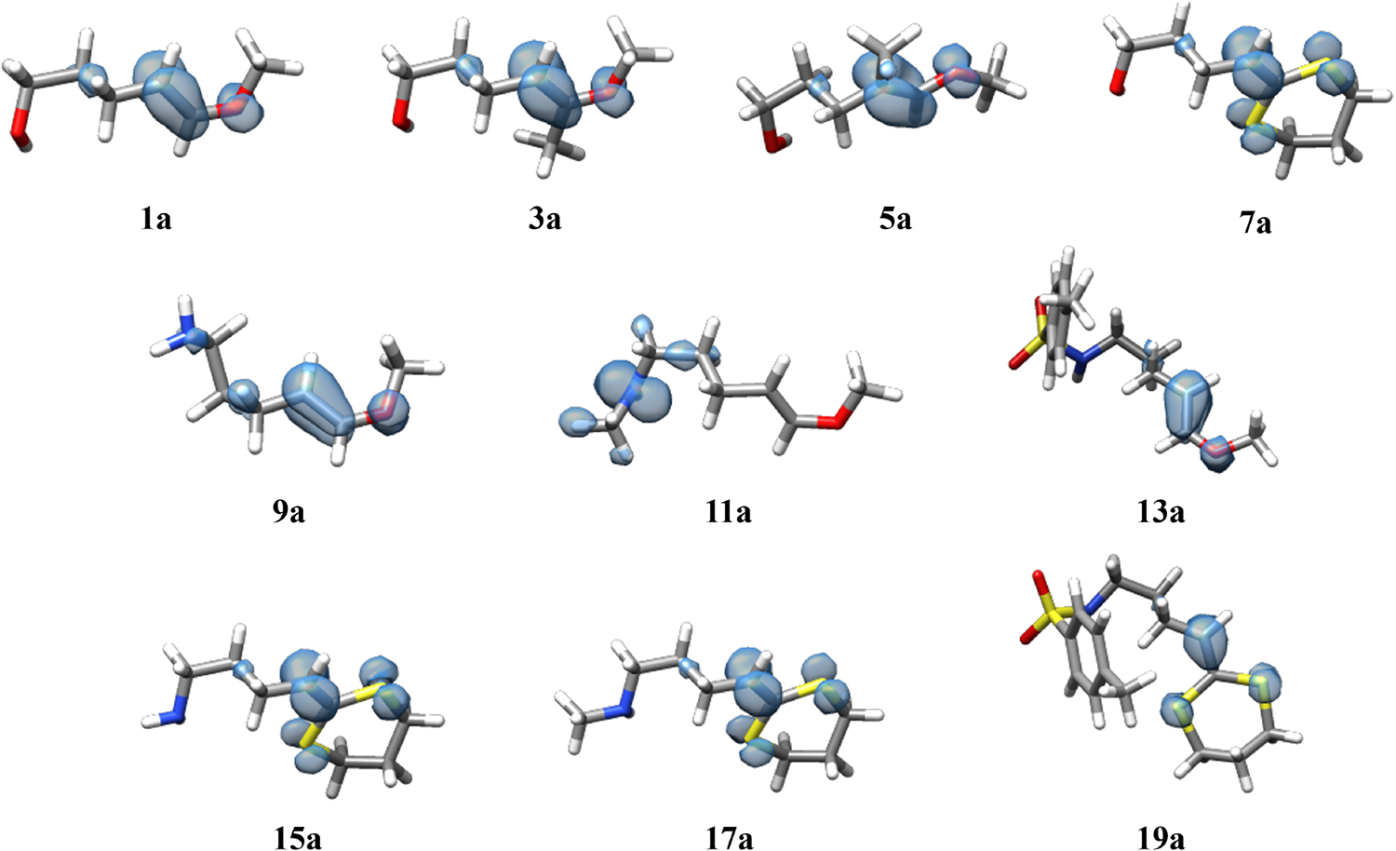
Spin density isosurfaces for cation radicals corresponding to oxidized substrates of Figure 2. The isosurfaces are plotted at values of 0.005 atomic units of electron density.

Trends in the computed oxidation potentials largely follow expectations based on the electroauxiliary group and the type of nucleophilic group (alcohol, amine, or sulfonamide) of the substrate. For the alcohol substrates, oxidation potentials decrease in order of substrates **1** > **3** > **5** ∼ **7**. Substrate **1** with an unsubstituted enol ether group has the highest oxidation potential, and substrates **3** and **5** with substituted enol ether groups have lower oxidation potentials. Substrates **7** and **1** are identical except for their electroauxiliary groups, and **7** with a ketene dithioacetal group (compared to enol ether) has the lower oxidation potential of the two substrates. For the amine and sulfonamide substrates with enol ether groups (**9**, **11**, and **13**), the different functional groups are spatially distant from the enol ether electroauxiliary, and one thus may expect the oxidation potentials of these substrates to be similar. This is the case for substrates **9** and **13**, which have similar oxidation potentials. However, for substrate **11**, which contains a secondary amine, the oxidation potential is significantly lower than these other two substrates by ∼0.2–0.3 V. The reason for this, as confirmed by DFT spin density analysis (subsequently discussed in Figure 4), is that the secondary amine group exhibits a lower oxidation potential compared to enol ether, and oxidation of substrate **11** is unique, as it occurs at the amine group. The last class of amine and sulfonamide substrates (**15**, **17**, and **19**) are those containing ketene dithioacetal electroauxiliary groups. All three substrates exhibit similar oxidation potentials, as oxidation of the ketene dithioacetal is largely unaffected by functional group differences for the amine/sulfonamide nucleophile. Furthermore, oxidation potentials of substrates **15**, **17**, and **19** are lower than values for substrates **9** and **13** by 0.2–0.4 V, given the lower oxidation potential of ketene dithioacetals compared to enol ethers.

A major conclusion from the computed oxidation potentials is determining whether the products of the intramolecular coupling reactions will be oxidatively stable under the reaction conditions. The first criterion is simply to evaluate whether the oxidation potential of the product is higher than that of the reactant. From Figure 3, it is observed that for all alcohol substrates, coupling products **2**, **4**, **6**, and **8** exhibit substantially higher oxidation potentials compared to their corresponding reactant precursors **1**, **3**, **5**, and **7**. On the other hand, this is not the case for the amine substrates; coupling products **10**, **12**, and **18** exhibit lower oxidation potentials than their reactant precursors **9**, **11**, and **17**. This is because the secondary (**10**) and tertiary (**12** and **18**) amines formed upon cyclization to the product have lower oxidation potentials compared to the initial electroauxiliary precursors (i.e., either enol ether or ketene dithioacetal). The sulfonamide substrates do not exhibit this “overoxidation” problem, as cyclic products **14** and **20** have substantially higher oxidation potentials compared to their precursors **13** and **19**. This overoxidation concern is why prior experimental work has largely employed sulfonamides (or imines^3^) rather than bare amine substrates for such anodic intramolecular coupling reactions.^2,4,6,8,11^

A “borderline” case is substrate **15**, for which cyclized product **16** has a computed oxidation potential that is only ∼0.2–0.3 V higher than the reactant substrate (Figure 3). Indeed, prior estimates suggest that the secondary amine of product **16** should be easier to oxidize than either the primary amine or ketene dithioacetal groups of substrate **15**, so that the product would be expected to be oxidatively unstable under reaction conditions.^11^ However, Moeller and co-workers have successfully employed substrates similar to **15** in anodic intramolecular coupling reactions while avoiding any overoxidation issues.^11^ The key reason for the success of this reaction is that substrates similar to **15** exhibit lower oxidation potentials than may be expected, experimentally measured to be ∼0.6–0.7 V vs Ag/AgCl. These authors rationalized the relatively low oxidation potential of the substrate as resulting from a “Nernstian shift” that occurs when the subsequent cyclization step following oxidation is very rapid.^11^ We have previously noted from calculations on similar substrates that computed “adiabatic” oxidation potentials can be lower than “vertical” oxidation potentials by 0.2–0.3 V, due to the cation radical changing the structure to a “folded” geometry with orbital interactions between oxidized electroauxiliary and nucleophile groups.^61,62^ Both of these effects have been neglected for the present oxidation potential computations, and Figure 3 values should thus be interpreted for a qualitative comparison. The final subtle point is that for borderline cases such as substrate **15**, constant voltage compared to constant current electrolysis may be preferable to control the potential and prevent product overoxidation, but this consideration is largely beyond the scope of our work.

#### 3.1.1 Spin Density Analysis of Oxidized Cation Radical Intermediates

As mentioned, the premise for the general reaction mechanism shown in Figure 1 for the anodic intramolecular coupling reactions is that the enol ether or ketene dithioacetal electroauxiliary group (rather than the nucleophile) is oxidized to a localized cation radical. Specifically, for those substrates in Figure 2 containing amine nucleophiles, this may not be the case, given the relatively low oxidation potentials of amines. To explore this question, we analyze the spin density of the cation radical intermediates, as computed from DFT, which qualitatively indicates the functional group of the substrate that was oxidized and where the resulting radical character is localized. Figure 4 depicts isosurfaces of the radical spin density for oxidized cation radicals corresponding to the ten different substrates of Figure 2. For oxidized substrates **1**, **3**, **5**, **9**, and **13**, the spin density clearly is localized on the enol ether group, indicating that this is the functional group that was oxidized. For oxidized substrates **7**, **15**, **17**, and **19**, the spin density clearly resides on the ketene dithioacetal group, indicating that this functional group was oxidized. The outlier is the cation radical for oxidized substrate **11**; for this substrate, the radical character is localized on the secondary amine group rather than the enol ether electroauxiliary. It was already discussed in the context of oxidation potentials in Figure 3 that substrate **11** was an outlier, and the spin density analysis in Figure 4 confirms the origin of this behavior. While nitrogen-centered radicals have utility in other electrosynthesis reactions and mechanisms,^63^ substrate **11** is seemingly not compatible with the general cation radical reaction mechanism proposed in Figure 1.

To conclude this section, the computed oxidation potentials (Figure 3) and spin density analysis (Figure 4) indicate which substrates of Figure 2 are potentially good candidates for anodic intramolecular coupling reactions. The conclusion is that substrates **1**, **3**, **5**, **7**, **13**, and **19** are all good candidates, given that products are expected to be oxidatively stable under reaction conditions and the reaction would likely proceed via the proposed scheme in Figure 1. This analysis is perfectly in line with the large body of work from the Moeller group, demonstrating successful intramolecular coupling reactions with similar substrates.^1,2,4,6,10,11,16,27^ Additionally, substrate **15** is also a good candidate, given experimentally demonstrated precedent,^11^ though this would have been difficult to predict a priori by computation alone, given the close oxidation potentials of the product and substrate. In the next section, we will downselect to four of these substrates and thoroughly investigate solvent effects on the free energy profiles for the cation radical cyclizations.

### 3.2 Cyclization Reactions of Cation Radical Intermediates: Role of Solvent in Stabilizing the Acidic Proton of Oxonium/Ammonium Groups

Based on the results/analysis of Section 3.1, as well as experimental precedent,^2,9,10,16^ we downselect to four substrates, namely, **1**, **7**, **13**, and **19**, to study the intramolecular cyclization reaction of cation radical intermediates following initial oxidation. The corresponding cyclization reactions for cation radical intermediates **1a**, **7a**, **13a**, and **19a** of these substrates are shown in Figure 5. These four substrates contain either alcohol (**1**, **7**) or sulfonamide (**13**, **19**) nucleophiles, which attack either enol ether or ketene dithioacetal-based cation radicals to form cyclic oxonium or ammonium intermediates. The proton on the cyclic oxonium or ammonium intermediates is highly acidic and will be lost/deprotonated in a subsequent step toward the final product formation (Figure 2). However, the stability of the protonated, cyclic oxonium, or ammonium intermediates shown in Figure 5 may play a key role in the product yield/selectivity of the target reaction. In a previous study,^62^ we computationally investigated the oxidative cyclization reaction of a tetramethoxyhexenol substrate similar to **1** and demonstrated that methanol solvent was crucial in driving the cyclization of the cation radical intermediate due to solvent complexation with the acidic proton. In this section, we seek to generalize the findings of ref 62 to a broader scope of substrates/solvent media, via a similar computation of cation radical cyclization reaction free energies.

**Figure 5:**
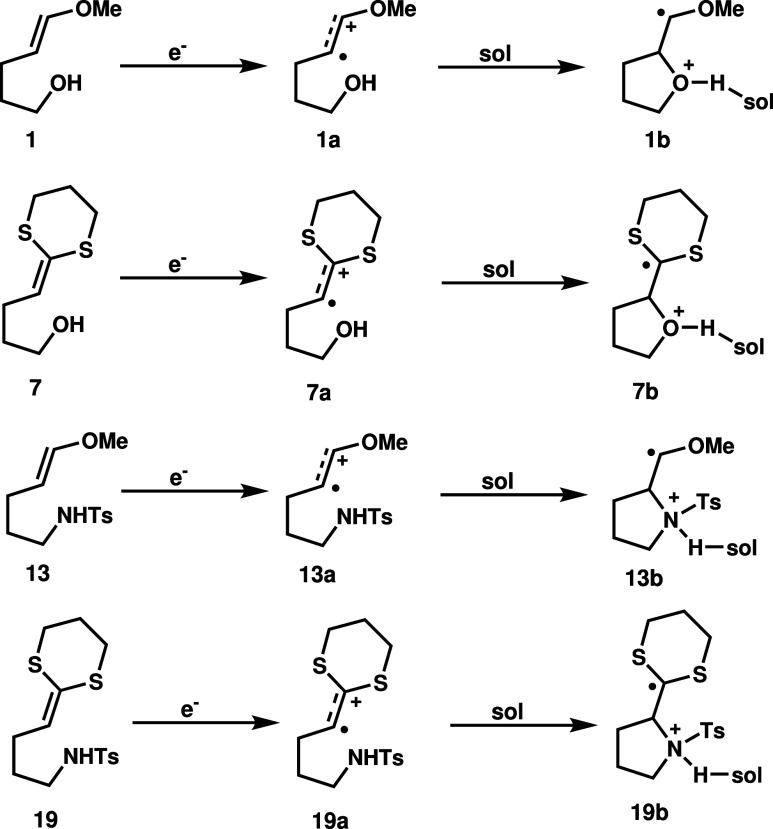
Cyclization reactions of cation radical intermediates for substrates **1**, **7**, **13**, and **19**. Following cyclization from the attack of an alcohol or sulfonamide nucleophile, the acidic proton of the oxonium or ammonium group is complexed by a solvent molecule denoted “sol” = methanol, tetrahydrofuran, acetonitrile, or dichloromethane.

DFT-based QM/MM-MD simulations in combination with free energy sampling were conducted to compute reaction free energies (and barriers) for cation radical species **1a**, **7a**, **13a**, and **19a** within solvents methanol (MeOH), tetrahydrofuran (THF), acetonitrile (ACN), and dichloromethane (DCM). The details of these computations are described in Section 2. Briefly, the QM/MM-MD simulations consisted of the cation radical species solvated in bulk solvent (2000 solvent molecules with periodic boundary conditions), with the solvent modeled at “MM” and the cation radical species modeled at “QM” (DFT) level of theory. In addition, we conducted separate QM/MM-MD simulations in which the closest solvent molecule that forms a hydrogen bond to the −OH or –NHTs group of the cation radical substrate is also included in the “QM” region. In the latter case, the solvent molecule included in the “QM” region can “complex” with the acidic proton of the oxonium/ammonium group upon intramolecular cyclization, as schematically depicted in Figure 5. As discussed in our previous work,^62^ such solvent complexation can substantially contribute to the thermodynamic driving force for the intramolecular cation radical cyclization. Covalent complexation (beyond simply hydrogen bonding) is only captured when the substrate/solvent molecules are both modeled with QM; thus, our comparative QM/MM-MD simulations with either zero or one solvent molecule in the QM region directly elucidate the solvent complexation contribution to the cation radical cyclization free energy.

The free energy profiles for intramolecular cyclization reactions of cation radicals **1a**, **7a**, **13a**, and **19a** are shown in Figure 6a–d, as computed in the four different solvents MeOH, THF, ACN, and DCM. Each free energy profile exhibits two minima separated by a free energy barrier, corresponding to uncyclized and cyclized cation radical substrates (e.g., **1a** and **1b** in Figure 6a). The reaction coordinate in the free energy profiles corresponds to the distance of the formed bond for the cation radical heterocycle, which is either *R*_C–O_ or *R*_C–N_ in the case of alcohol or sulfonamide trapping. The cyclized products correspond to bond distances of *R*_C–O_ ∼ 1.5 Å or *R*_C–N_ ∼ 1.5 Å, while the free energy minima for the uncyclized cation radical substrate are located near *R*_C–O_ ∼ 2.5 ± 0.2 Å or *R*_C–N_ ∼ 2.5 ± 0.2 Å, depending on the substrate. Note that the free energy minima of the uncyclized cation radical substrates correspond to “folded” or “pre-cyclized” geometries rather than extended linear structures, and the minimum results from orbital interaction between the lone electron pairs of the nucleophile and the cation radical electrophile. The nature of these “folded” geometries were discussed extensively in our previous work^62^ and will not be extensively discussed here.

**Figure 6:**
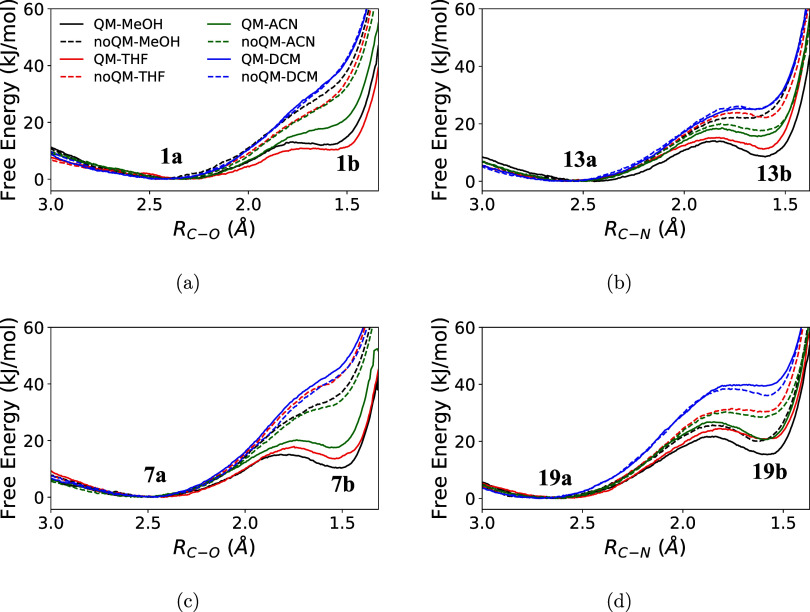
Free energy profiles for the cyclization reactions of cation radical substrates (a) **1a**, (b) **13a**, (c) **7a**, and (d) **19a** as computed from QM/MM-MD simulations in bulk solvents. The reaction coordinate is the cyclic bond distance, *R*_C–O_ for substrates **1a** and **7a** and *R*_C–N_ for substrates **13a** and **19a**. Free energy profiles are computed for substrates within bulk solvents methanol (MeOH), tetrahydrofuran (THF), acetonitrile (ACN), and dichloromethane (DCM), corresponding to black, red, green, and blue curves, respectively. Profiles depicted as solid lines are computed from QM/MM-MD simulations with the substrate and one solvent molecule in the QM region, while profiles depicted as dashed lines are from simulations with only the substrate (no solvent) in the QM region.

The initial observation from Figure 6 is that the DFT-based QM/MM-MD simulations predict that the cyclized cation radical structures (**1b**, **7b**, **13b**, and **19b**) are higher in free energy than their corresponding uncyclized structures (**1a**, **7a**, **13a**, and **19a**), so that the intramolecular cyclization reactions would appear unfavorable. This is the predicted trend regardless of the substrate, solvent, or computational details (e.g., zero vs one solvent in QM region). However, this prediction is most likely incorrect/unphysical and rather is a failure of DFT for predicting the relative energetics of cation radical intermediates (as resulting from self-interaction error).^32–36^ In Table S3 in the Supporting Information, we provide higher level MP2/cc-pVTZ benchmarks for the cyclization reaction free energies of substrates **1a**, **7a**, **13a**, and **19a** as computed in implicit solvent (CPCM). As shown in Table S3, the PBE0-D3/6-31G* level of theory utilized for the QM/MM simulations underestimates the stability of the cyclized cation radical structures as compared to MP2/cc-pVTZ, by ∼5–20 kJ/mol depending on the substrate. The error is further exaggerated for DFT calculations employing larger basis sets (e.g., PBE0-D3/6-311G*), given that a combination of density functionals with smaller basis sets may often benefit from error cancellation for such reactions.^62^ The takeaway is that cyclization reaction thermodynamics presented in Figure 6 are likely systematically too unfavorable by ∼5–20 kJ/mol, and interpretation should focus on relative trends in the predicted solvation effects on the reaction free energies (which are expected to be robust in spite of any DFT errors).

As indicated by the free energy profiles in Figure 6, solvent complexation with the acidic proton of the oxonium/ammonium group of the cyclized cation radical substrates provides a major stabilization/driving force for the reactions. This is evidenced by comparing free energy profiles from QM/MM-MD simulations with no solvent molecules in the QM region (dashed curves) to profiles from QM/MM-MD simulations with the close/complexing solvent molecule in the QM region (solid curves). As discussed, only when this solvent molecule is included in the QM region can it covalently complex to the acidic proton (when this solvent molecule is modeled as “MM”, the interaction is purely electrostatic and van der Waals (VDWs)). All four reactions of cation radicals **1a**, **7a**, **13a**, and **19a** exhibit similar solvent effects. The cyclization reactions are least favorable/facile within DCM solvent, which exhibits little-to-no ability to complex/stabilize the acidic proton of cyclized oxonium/ammonium groups. In fact, the free energy profiles for the cyclization reactions within DCM are very similar to those for QM/MM-MD simulations with/without the solvent molecule in the QM region (solid blue vs dashed blue curves in Figure 6), indicating there is no covalent complexation between DCM and the acidic proton (and the interaction is purely electrostatic/VDWs). When the cation radical reactions occur in the other solvents, MeOH, THF, or ACN, the cyclization reactions are much more favorable/facile, as indicated by the shift from the solid blue curve (DCM) to solid black/red/green curves (for MeOH/THF/ACN, respectively).

The net solvent effect on the cation radical reaction thermodynamics is substantial. For substrates **13a** and **19a** (Figure 6b,d) with the sulfonamide nucleophile coupling to either enol ether or ketene dithioacetal cation radicals, the modulation from switching from DCM (solid blue curves) to more polar MeOH solvent (solid black curves) is ∼15–20 kJ/mol in driving/facilitating the intramolecular cyclization reaction; there is a similar but somewhat smaller shift for THF (solid red curve) and ACN (solid green curves) solvents. For substrates **1a** and **7a** (Figure 6a,c) with the alcohol nucleophile coupling to either enol ether or ketene dithioacetal cation radicals, the modulation from switching from DCM (solid blue curves) to MeOH solvent (solid black curves) is ∼30–35 kJ/mol in driving the intramolecular cyclization reaction (with a similar shift for THF (solid red curves) and a slightly smaller shift for ACN (solid green curves) solvent). *The implication is that such solvent effects may be crucial in facilitating the desired anodic intramolecular coupling chemistry.* Recall that based on the MP2/cc-pVTZ implicit solvent benchmark calculations in Table S3, DFT underpredicts the cyclization reaction thermodynamics by ∼5–20 kJ/mol. In light of this DFT error, Figure 6 suggests that the intramolecular cation radical cyclization reactions are likely thermodynamically feasible within polar MeOH, THF, and ACN solvents, but not within the nonpolar and noncomplexing DCM solvent.

The solvent effects can be understood/decomposed as electrostatic and covalent complexation contributions by analyzing the different QM/MM-MD free energy profiles. The interpretation is similar for all four substrates, so for brevity, we focus on the cyclization reaction of substrate **7a** to **7b** (Figure 6c). The free energy profiles drawn as dashed curves are those in which all solvent molecules were modeled at the “MM” level of theory, so that interactions between the cation radical substrate and solvent are purely electrostatic/VDWs (but no covalent or charge transfer character). In this case, the relative stability of cyclic cation radical **7b** increases with increasing solvent dielectric constant, in the order DCM ∼ THF < MeOH < ACN (blue, red, black, and green dashed curves, respectively). As the solvent dielectric increases, there is enhanced electrostatic stabilization of the oxonium cation charge in the cyclic **7b** product. While both **7a** reactant and **7b** product have the same net charge, the cation charge is more localized (i.e., on the oxonium group) in the cyclic **7b** product, which is why the solvent dielectric (electrostatics) modulates the product stability relative to the reactant.

For each solvent, comparing the dashed and solid curves in Figure 6c indicates the covalent solvent complexation to the oxonium cation group, which further stabilizes the cyclic **7b** product. To recall, the solid curves are free energy profiles computed from QM/MM-MD simulations in which the close/complexing solvent molecule is treated at the “QM” level of theory, so that it exhibits full quantum mechanical (i.e., covalent interaction, charge transfer interaction, etc.) interaction with the cation radical substrate. As previously mentioned, as DCM solvent has little-to-no Lewis base character and cannot complex to the proton on the oxonium cation group, the two free energy profiles (blue solid and dashed curves) computed in DCM solvent are nearly identical. ACN, being a weak Lewis base (but stronger than DCM), forms a partially covalent complex with the proton of the oxonium cation, and thus, there is a significant increase in the stability of the cyclic **7b** product predicted in the solid green compared to the dashed green free energy profile (i.e., when covalent complexation is incorporated). MeOH and THF are the strongest Lewis bases of the four solvents and thus provide the most substantial stability enhancement of the cyclic **7b** product via covalent/complexation of the oxonium cation proton. Note that comparing the free energy profiles within ACN and THF provides a very clear and direct elucidation of electrostatic vs covalent complexation stabilization. The dashed green (ACN) and red (THF) curves predict that **7b** is relatively more stable in ACN when only electrostatics is taken into account (since ACN has a higher dielectric constant). However, the solid green (ACN) and red (THF) curves predict that **7b** is relatively more stable in THF when full quantum mechanical interactions of the solvent are included, given the Lewis base character of THF and the ability to complex the acidic oxonium proton. For the **7a**-to-**7b** cation radical cyclization (as well as **1a** to **1b** in Figure 6a), the Lewis base character of the solvent is the more dominant effect in stabilizing the oxonium proton of the cyclic product.

In Figure 7, we show a snapshot from the QM/MM simulations of reaction **1a** ↔ **1b** in methanol solvent, depicting methanol complexation to the acidic proton of the cyclic cation radical **1b** species. As observed, the acidic proton is shared between the oxygen atoms of the substrate and methanol, with O–H bond distances of ∼1.0 and 1.3 Å, respectively. In a previous work investigating a similar (but slightly different) cyclic oxonium cation radical substrate, we noted a similar shared proton motif, but with somewhat different bond lengths; indeed whether the acidic proton is closer to substrate or methanol oxygen atoms depends sensitively on the nature of the substrate.^62^ Cyclic oxonium substrates **1b** and **7b** exhibit typical O–H distances of 1.3–1.4 Å between the acidic proton and the oxygen atom of the complexed methanol solvent molecule. On the other hand, cyclic substrates **13b** and **19b** with protic ammonium cations are less acidic and exhibit (longer) O–H distances of 1.6–1.7 Å between the acidic proton and the oxygen atom of a complexed methanol solvent molecule. These structural differences correlate with differences in the magnitude of solvation effects on the free energy profiles in Figure 6 observed for MeOH and THF solvents (and ACN to a lesser extent). Taking as an example the cyclization reaction of **19a** to **19b** in MeOH or THF solvent (black and red curves in Figure 6d), the difference in **19b** product stability between dashed and solid curves is ∼5–10 kJ/mol. This ∼5–10 kJ/mol difference reflects the stabilization of MeOH or THF Lewis base complexation with the protic ammonium cation group (i.e., beyond the electrostatic contributions). In contrast, the comparative difference was much larger ∼20–25 kJ/mol for the discussed cyclization of substrate **7a** to **7b** (Figure 6c) with solvent complexation of the protic oxonium cation group.

**Figure 7:**
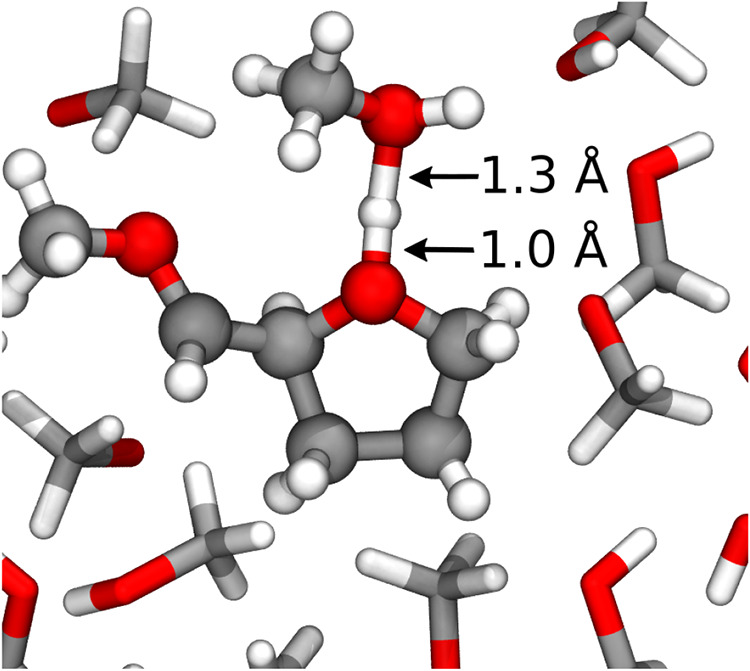
QM/MM simulation snapshot of the cyclized cation radical **1b** structure in methanol solvent. The acidic proton is complexed to a methanol solvent molecule, with a bond distance of ∼1.3 Å to the methanol oxygen atom.

The overall/takeaway conclusion from this section is that solvent effects are crucial for facilitating the cation radical cyclizations of substrates **1**, **7**, **13**, and **19**. Substantial driving force is provided by the more basic THF and MeOH solvents, modulating the cyclization thermodynamics by up to ∼30–35 kJ/mol for the oxonium cation radicals and ∼15–25 kJ/mol for the ammonium cation radicals, compared to baseline reactions in DCM solvent. We note that the related free energy profiles discussed in Section 3.3 demonstrate that these quantitative predictions are converged with respect to the number of solvent molecules partitioned in the QM region. In the next Section, we will investigate the activation barriers for deprotonation of the acidic cyclic cation radical species.

### 3.3 Deprotonation of the Protic Oxonium Group of Cyclic Cation Radical Substrate **1b**

The thermodynamics and kinetics of cation radical cyclization, as analyzed in Section 3.2, are likely a crucial factor governing the yield/selectivity of proposed anodic intramolecular coupling reactions, such as those considered in Figures 1 and 2. This is because there are often numerous competing side reactions that the cation radical can undergo (e.g., solvent nucleophilic attack^62^), and the yield/selectivity of the target cyclization pathway will be dictated by kinetic competition among these possible reaction pathways. But the kinetics of subsequent mechanistic steps following the cation radical cyclization may also be important in dictating the overall reaction yield/selectivity. Indeed, Moeller and co-workers have demonstrated unequivocally that the rate of second oxidation is an important determiner of product yield/selectivity in these classes of anodic intramolecular coupling reactions.^2–4,6,7^ In this section, we provide a more detailed analysis of the mechanistic steps following cation radical cyclization, focusing on substrate **1**.

Figure 8 provides a more detailed picture of the reaction mechanism for anodic cyclization of substrate **1**, explicitly considering the second oxidation and deprotonation steps following the cation radical cyclization. Substrate **1** is first oxidized at the anode to **1a**, which undergoes cyclization to **1b**; the kinetics and thermodynamics of this mechanistic step depend on solvent, as explicitly discussed in Figure 6 of Section 3.2. Upon cyclization to **1b**, the proton of the oxonium cation group is highly acidic, and deprotonation of **1b** to solvent (e.g., MeOH, THF, etc.) to form **1c** will be thermodynamically favorable (even in the absence of a base). If the kinetics of transformation **1a** to **1b** to **1c** is rapid such that **1c** still resides at the anode surface (i.e., with an electron transfer distance of ∼1 nm), oxidation via heterogeneous electron transfer of **1c** to **1e** is both thermodynamically favorable and kinetically rapid, given the expected moderate-to-large overpotential for this electron transfer at the working electrode (since oxidation potential of **1c** is typically lower than that of substrate **1**). Once cation **1e** is formed, it is expected to react very rapidly (e.g., barrierless kinetics) with solvent via nucleophilic attack to form stable product **1f**.

**Figure 8:**
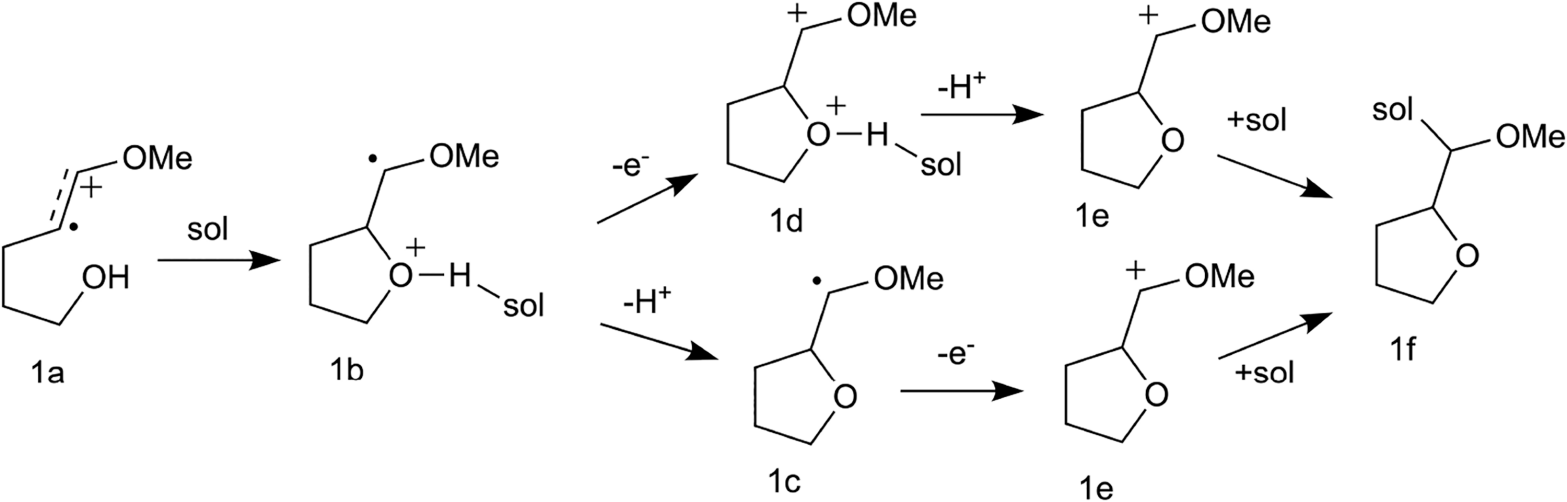
Cyclization reaction of substrate **1** depicting different mechanistic pathways for deprotonation and second oxidation steps. Substrate **1** is oxidized at the anode to **1a**, which undergoes cyclization to **1b**. The subsequent second oxidation and deprotonation steps may then proceed sequentially in various orders: Deprotonation of **1b** to **1c** may precede the second oxidation to **1e**; however, if deprotonation kinetics are slow relative to electron transfer, **1b** may first oxidize to dication **1d**, followed by deprotonation to **1e**. Once **1e** is formed, solvent “sol” trapping of the cation will give stable product **1f**.

However, if the deprotonation kinetics of **1b** is relatively slow, two other different pathways could occur instead. First, **1b** could desorb/diffuse away from the anode surface and deprotonate to **1c** homogeneously, in which case radical intermediate **1c** cannot undergo second oxidation via heterogeneous electron transfer (i.e., too far from the electrode). The second oxidation of **1c** to **1e** would then proceed homogeneously via a disproportionation reaction

3 1c+1a↔1e+1

Assuming that the working electrode (anode) is held at a potential equal to the oxidation potential of substrate **1**, the heterogeneous electron transfer and homogeneous disproportionation for the second oxidation of **1c** will exhibit equivalent thermodynamics. However, the kinetics would be distinctly different; disproportionation would follow second-order kinetics in electrogenerated intermediates, while the heterogeneous electron transfer would be first-order kinetics, with the rate increasing exponentially with overpotential (i.e., Butler–Volmer).^28^ Since radical intermediate **1c** would be prone to side reactions, the specific mechanism/pathway could affect overall product yield/selectivity due to differing lifetimes of radical **1c** and thus its susceptibility to side reactions.

The other possibility is that the second oxidation occurs at the electrode before (or at least initiates) deprotonation. This is the pathway shown in Figure 8, in which **1b** is oxidized at the anode to dication **1d**. Dication **1d** will then deprotonate to cation **1e**, and attack of the solvent will give product **1f**. Note that dication **1d** may not be a stable species, and deprotonation would likely be concurrent or coupled with the oxidation (i.e., proton-coupled electron transfer).^28^ The purpose of showing the **1d** intermediate in Figure 8 is simply to distinguish this pathway from the case in which the deprotonation from **1b** to **1c** occurs sequentially before the second oxidation.

In this section, we computationally investigate the deprotonation kinetics (e.g., activation free energies) for the deprotonation of **1b** to **1c** by solvent. We focus on three solvents MeOH, THF, and ACN and do not consider DCM based on the results of Section 3.2. Given that cation radicals exhibit superacidic behavior,^64,65^ solvents such as MeOH, THF, and ACN can indeed function as proton acceptors, forming corresponding conjugate acids: methyloxonium,^66^ tetrahydrofuranoxonium,^67^ and acetonitrilium.^68^ However, these conjugate acids are relatively unstable unless stabilized through the formation of solvent dimers/complexes, where the excess proton is shared equally between two or more solvent molecules.^69^ To investigate the deprotonation kinetics, we conduct DFT-QM/MM free energy simulations for the reaction **1a** ↔ **1b** ↔ **1c** within the bulk solvents MeOH, THF, and ACN. The methodological details of these simulations have been described in Section 2. The motivation for computationally constructing the full free energy profile for **1a** ↔ **1b** ↔ **1c**, including both the cyclization and deprotonation steps, is to explicitly evaluate whether­(protonated) cyclic cation radical species **1b** is a metastable intermediate.

Figure 9 shows the free energy profiles encompassing the cyclization and deprotonation steps **1a** ↔ **1b** ↔ **1c** for cation radical substrate **1a**, in MeOH, THF, and ACN bulk solvents. As discussed in Section 2, two-dimensional (2D) free energy profiles are computed considering both the cyclization coordinate (“*R*_C–O_” C–O distance, *y*-axis) and the proton transfer coordinate (“*R*_PT_”, *x*-axis). The proton transfer coordinate *R*_PT_ is defined in Section 2; a negative value −1 ≤ *R*_PT_ ≤ 0 indicates that the proton has a shorter bond distance to the substrate oxygen atom compared to the solvent acceptor atom (and vice versa for positive values 0 ≤ *R*_PT_ ≤ 1). Three species **1a**, **1b**, and **1c** correspond to local free energy minima on the 2D profiles: Species **1a** corresponds to a local free energy minimum observed near *R*_PT_ = −0.75 Å and *R*_C–O_ = 2.25 Å; species **1b** corresponds to a local free energy minimum observed near *R*_PT_ = −0.25 Å and *R*_C–O_ = 1.6 Å; and species **1c** corresponds to a local free energy minimum near *R*_PT_ = 0.5 Å and *R*_C–O_ = 1.5 Å. In Figure 9, two different QM/MM free energy profiles are shown for the reaction within each solvent system: Profiles labeled “1 ACN/THF/MeOH” were computed with cation radical substrate **1a** and the solvent molecule acting as the proton acceptor within the QM region, and remainder of the solvent in the MM region; profiles labeled “2 ACN/THF/MeOH” included two solvent molecules, the proton acceptor solvent and an additional proximal solvent molecule, in the QM region in addition to substrate **1a**. This comparison mirrors that of Section 3.2 and, as will be discussed, elucidates the role of a second solvent molecule in complexing with the acidic solvent species following deprotonation of **1b** to **1c**.

**Figure 9:**
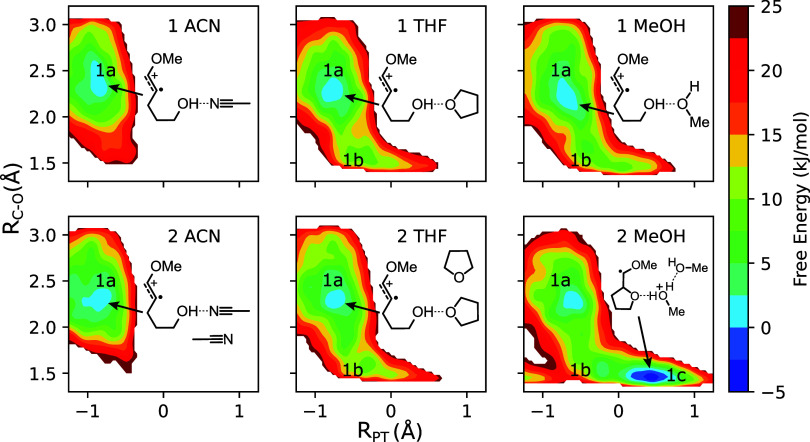
2D free energy profiles for cyclization and deprotonation of cation radical **1a** to **1b** to **1c**. Free energy profiles are computed for the reaction in bulk ACN, THF, and MeOH solvents and correspond to QM/MM simulations that contain either one “1 ACN/THF/MeOH” or two “2 ACN/THF/MeOH” solvent molecules modeled at the QM level (the remainder of the solvent is modeled at the MM level). Additionally, within each plot, the chemical structure corresponding to the lowest free energy configuration on the free energy surface is shown.

We initially discuss the reaction free energy profiles within MeOH solvent, as illustrated in Figure 9. The free energy profile labeled “2 MeOH” clearly exhibits three local minima corresponding to **1a**, **1b**, and **1c** species indicated by light/dark blue regions on the free energy profile and separated from each other by activation barriers (green regions). As mentioned, the “2 MeOH” label indicates that two methanol solvent molecules were modeled at the QM level within this free energy simulation (the importance of this choice will be discussed). The global minimum (dark blue region) in the profile located at *R*_PT_ = 0.5 Å and *R*_C–O_ = 1.5 Å corresponds to the deprotonated **1c** species, with the labile proton being “shared” between the two QM solvent methanol molecules, as indicated schematically in the figure inset. This conforms with the expectation that it is thermodynamically favorable for strongly acidic cation radical species **1b** to deprotonate to solvent (at least of moderate basicity). Of key note is the finite activation barrier of 5–10 kJ/mol separating the free energy minimum for species **1c** from the free energy minimum for the protonated **1b** species. The presence of this (albeit moderate) activation barrier definitively indicates that the acidic protonated **1b** species is a well-defined reaction intermediate, with a finite kinetic lifetime. As will be discussed, the lifetime of species **1b** is strongly solvent/environment-dependent and may be an important reason for solvent-modulated changes in product selectivity/yield for the electrosynthesis reaction.

Comparing the free energy profile “2 MeOH” to the profile “1 MeOH” in Figure 9 explicitly indicates the role of the second methanol solvent molecule in the deprotonation reaction. As mentioned, the “1 MeOH” profile is computed from QM/MM simulations with only one methanol molecule (e.g., the proton acceptor) and the **1a** substrate in the QM region. Thus, when the proton is transferred to methanol, it must stay localized on the single solvent molecule, without complexing to another nearby solvent molecule (since the remainder of the solvent is modeled at the MM level). Because of this difference in modeling, there is no corresponding free energy minimum for the deprotonated **1c** species in the “1 MeOH” free energy profile. Clearly, the second QM methanol molecule thus makes a crucial contribution to the thermodynamic driving force for deprotonating **1b** to **1c** (“2 MeOH” profile in Figure 9), via stabilizing the solvated excess proton. When the primary methanol solvent molecule accepts the acidic proton from **1b**, the other proton on this methanol molecule becomes acidic and is partially transferred/shared to the second methanol molecule. Specifically at the **1c** minimum geometry (labeled in Figure 9), the secondary proton on the primary methanol is equally shared between both methanol molecules, with O–H bond distances of ∼1.2 Å to both oxygen atoms of the two methanol molecules. In this way, the excess proton charge is “delocalized” among the (MeOH)_2_H^+^ methanol dimer complex, providing additional stability and requisite thermodynamic driving force for deprotonation of **1b** to **1c**. This is reminiscent of proton delocalization in water, and indeed, the geometry of the shared proton complex between the two methanol solvent molecules and **1c** species is reminiscent of the well-known “Zundel” motif for the H_5_O_2_^+^ species of a solvated proton in water.

The other free energy profiles in Figure 9 correspond to the reaction within either ACN or THF bulk solvents. These profiles are distinctly different from the “2 MeOH” profile, in that there is no free energy minimum observed for the deprotonated **1c** species. Furthermore, unlike the free energy profiles for methanol solvent (“1 MeOH” and “2 MeOH”), there is no significant difference between the free energy profiles with different numbers of QM solvent molecules (i.e., “1 THF” and “2 THF” profiles are qualitatively identical). The lack of a free energy minimum observed for the **1c** species within THF and ACN solvents implies that these solvents are not nearly as effective as MeOH at deprotonating cation radical **1b** to **1c**. While all three solvents are effective hydrogen bond acceptors via their electronegative oxygen/nitrogen atoms, MeOH is a protic solvent, whereas THF and ACN are not. The unique ability of MeOH to facilitate deprotonation of **1b** to **1c** thus arises from its protic nature, with the primary methanol molecule donating a proton to the secondary methanol molecule and simultaneously accepting a proton from the cation radical oxonium group of species **1b**. This allows the secondary methanol molecule to directly stabilize the proton acceptor methanol at the proton transfer transition state. This ability to participate in multiple hydrogen bonds simultaneously is the feature that sets MeOH apart from the other solvents in its ability to facilitate the deprotonation of **1b** to **1c**.

The takeaway from comparing the free energy profiles in Figure 9 to those in Figure 6 of Section 3.2 is that the solvents ACN, THF, and MeOH modulate the cyclization step (e.g., **1a** to **1b**) and deprotonation step (e.g., **1b** to **1c**) of the anodic coupling reactions to different degrees. All three solvents ACN, THF, and MeOH are effective at promoting the cyclization reaction for the various cation radical substrates in Figure 6, as they can form complexes with the acidic protons of the oxonium or ammonium groups of the cyclic cation radicals. However, formation of solvent/cyclic cation radical complexes (**1b** in Figure 9) does not guarantee facile deprotonation to the solvent. For example, comparing the **1a** ↔ **1b** ↔ **1c** reaction in THF and MeOH (Figures 6 and 9), the THF and MeOH solvents are nearly equally effective at driving the cyclization of **1a** to **1b**, via complexation of the acidic proton of **1b** with the electronegative oxygen atom on the THF or MeOH solvent molecule. However, deprotonation of **1b** to **1c** is much more facile within MeOH compared to THF for the reasons discussed. This implies that the lifetime of the acidic **1b** cyclic species will be different within the different solvents, exhibiting a longer lifetime in THF and a shorter lifetime in MeOH (due to rapid deprotonation of **1b** to **1c**), with possible implications for the electrosynthesis reaction outcome.

The free energy profiles depicted in Figure 9 provide a mechanistic rationale for solvent modulation of product yield/selectivity in anodic intramolecular coupling reactions that involve acidic cyclic cation radical intermediates. We note that solvent modulation of product yield/selectivity in such reactions has been extensively documented by the Moeller group.^1,2,9,10,12,16^ Admittedly, solvent effects in such anodic coupling reactions are complex; the solvent dictates the substrate’s affinity for the electrode surface as required for initial (and secondary) electron transfer, and additionally, the solvent may be involved in unwanted side reactions including trapping of cation radical intermediates. However, the free energy profiles in Figure 9 indicate a direct role of solvent in mediating the cyclization step **1a** to **1b** and deprotonation step **1b** to **1c** required for the success of the intramolecular coupling reaction. Effective solvents should have some Lewis basic character (e.g., MeOH, THF, ACN) to facilitate complexation and stabilization of the substrate acidic proton following cyclization, which provides the important thermodynamic driving force for cation radical cyclization (Figure 6). However, in THF and ACN solvents, deprotonation of the cyclized cation radical species may be slow and kinetically limiting to the full reaction mechanism. If deprotonation is relatively slow, the protonated intermediate (e.g., **1b**) may first diffuse out of the electrical double layer and away from the anode surface, at which point any second oxidation step would have to occur homogeneously via disproportionation. This would impact the lifetimes of reactive radical intermediates (e.g., **1c**) and thus may influence the propensity to unwanted side reactions. Clearly, a base could be added to the electrolyte to facilitate this deprotonation step, but addition of a base could have additional unintended consequences. In contrast, methanol solvent is very effective at both promoting cyclization and facile deprotonation (“2 MeOH” profile, Figure 9); in methanol, the transformation, e.g., **1a** → **1b** → **1c**, is rapid, so that intermediate **1c** may still reside in the anodic double layer and undergo heterogeneous second oxidation to **1e** to complete the reaction pathway. These computational results provide additional mechanistic understanding for why methanol has often been found to be a very effective solvent for anodic intramolecular coupling reactions.^1,2,9,10,12,16^

We conclude with a very brief discussion of the second possible reaction pathway depicted in Figure 8, for which the second oxidation precedes deprotonation. In this postulated mechanism, the protonated, cyclic cation radical species **1b** would undergo heterogeneous electron transfer to dication **1d**, followed by spontaneous deprotonation to **1e**. This postulated pathway could potentially be relevant/important if deprotonation **1b** to **1c** is relatively slow, as may be the case for certain solvents (discussion above). Table S4 in the Supporting Information reports computed second oxidation potentials for protic cyclic cation radical species **1b**, **7b**, **13b**, and **19b** shown in Figure 5. In these calculations, the oxidation potential was computed for the cyclic cation radical substrate complexed to a methanol solvent molecule, as solvent complexation is necessary to stabilize the protonated cyclic structure (Figure 6). As observed in Table S4, vertical second oxidation potentials for **1b**, **7b**, **13b**, and **19b** are systematically lower than the first oxidation potentials of the corresponding substrates (Figure 3) by ∼0.3–0.5 V. Thus, the cyclization coincident with solvent complexation leads to a sufficiently distonic cation radical electronic structure that the second oxidation of the radical is more thermodynamically favorable than the initial oxidation of the neutral substrate. Table S4 also indicates that the adiabatic second oxidation potentials are even lower still (more thermodynamically favorable), given significant geometric relaxation of the dication following oxidation; in the case of substrates **1b** and **7b**, the second oxidation is electrodissociative, with spontaneous proton transfer to the methanol solvent (Table S4).

The takeaway is that both pathways postulated in Figure 8 are predicted to be thermodynamically accessible at the working electrode potential, and branching between pathways will be dictated by kinetics. The relative kinetics for the pathways will depend on solvent (i.e., deprotonation rates) and electrolysis conditions such as overpotential (i.e., electron transfer rates). Prediction of the electron transfer kinetics is complex and beyond the scope of this work and would require, e.g., consideration of how outer-sphere reorganization energies differ for monovalent/divalent charge states and are modulated within the electrical double layer.^70,71^ Regardless, a seemingly robust conclusion from this work is the utility of methanol solvent in facilitating both the cyclization and deprotonation steps (Figures 6 and 9) to minimize lifetimes of radical intermediates (e.g., **1c**) as relevant to side reactions.

## 4 Conclusion

Our computational study has characterized reaction free energies and activation barriers for the fundamental microscopic steps of specific classes of anodic intramolecular coupling reactions that proceed through acidic cyclic cation radical intermediates. While the primary consideration for successful coupling reactions is the compatibility of the electroauxiliary group and nucleophile,^1,2,4,6,10,11,16^ the highly reactive nature of the electrogenerated intermediates (cation radicals, radicals, etc.) entails that they are prone to side reactions, and reaction conditions that modulate the relative kinetics can play a major role in determining product yield/selectivity. For this class of reactions, solvent effects may manifest in numerous ways, including unwanted side reactions involving solvent nucleophilic trapping of cation radical intermediates.^16^ In this work, we have focused on elucidating solvent effects on the direct/intended reaction pathway, including the thermodynamic contribution of the solvent in driving the cation radical cyclization step, and the efficacy of the solvent for deprotonating the acidic cyclic cation radical intermediate to precede the secondary oxidation step.

We have utilized DFT-QM/MM molecular dynamics simulations to predict free energy profiles for the cyclization and deprotonation steps of the electrogenerated cation radical intermediates for various substrates in MeOH, THF, ACN, and DCM solvents. A key finding is that for both oxonium and ammonium cyclic cation radical intermediates (formed via trapping by alcohol or sulfonamide nucleophiles), there is a substantial thermodynamic driving force for cation radical cyclization that arises from solvent stabilization of the acidic proton of the oxonium or ammonium group. The more basic THF and MeOH solvents provide the most favorable driving force, modulating the cyclization thermodynamics by up to ∼30–35 kJ/mol for the oxonium cation radicals and ∼15–25 kJ/mol for the ammonium cation radicals, compared to baseline reactions in DCM solvent. Given that these solvent shifts are on par or larger than the innate cyclization reaction thermodynamics (i.e., as computed at the MP2/cc-pVTZ/CPCM level in Table S3), the choice of solvent plays a crucial role in promoting/driving the cation radical cyclization step. We note that these conclusions about relative solvent effects are expected to be robust, despite the noted errors from DFT in predicting absolute reaction thermodynamics. Further characterization/analysis of the DFT-QM/MM simulations (i.e., comparing “1 solvent” vs “2 solvent” molecules in the QM region) decomposed the solvent effects into “basic” and “electrostatic” contributions. For the cyclic oxonium cation radicals, the primary “basic” effect is the electronegative oxygen (MeOH, THF) or nitrogen (ACN) atom complexing the acidic proton. Electrostatic forces make an important contribution to stabilizing the cyclic ammonium cation radicals, with reaction free energies modulated by solvent dielectric strength, and an additional contribution from covalent complexation with the acidic proton. An additional conclusion from our computed free energy profiles is that methanol is much more effective than the other solvents at deprotonating the cyclic cation radical intermediate, which likely enables the required second oxidation to occur heterogeneously at the anode surface, before the substrate diffuses out of the electrical double layer.

However, the practical choice of solvent must balance other considerations as well, including (but not limited to) the propensity of the solvent to participate in unwanted side reactions with the substrate. Outside of electrochemical stability considerations, nucleophilic solvents may participate in side reactions involving nucleophilic attack on the cation radical intermediate. Despite the seemingly desirable properties of methanol discussed above for facilitating the direct intramolecular cyclization reaction steps, a potential concern is that methanol may trap the cation radical intermediate and thus outcompete the intended intramolecular coupling; indeed, this was a motivation for tuning methanol solvent concentration in numerous prior experimental studies.^10,16,18,27^ In this regard, it is important to note that there is a rate order difference between intramolecular nucleophilic attack and solvent nucleophilic attack to the cation radical intermediate. In recent work,^62^ we demonstrated on the basis of computational predictions that for a related intramolecular cyclization reaction involving alcohol attack of an enol ether-based cation radical, the intramolecular attack followed overall second-order kinetics (first order in methanol concentration), while intermolecular solvent attack followed overall third-order kinetics (second order in methanol concentration). These rate orders differ from the otherwise intuitive assumption of first- and second-order kinetics, respectively, and result from the requisite driving force from solvent complexation to the acidic proton of the cyclic oxonium cation radical intermediate (in the case of intermolecular attack, the methanol nucleophile upon bond formation has an acidic proton that must be complexed by a secondary methanol molecule). These rate order differences are particularly important given that the anodic coupling reactions occur within the anodic double-layer environment, which is highly concentrated in ions, and solvent mobility is restricted;^16^ for these reasons, methanol may often be an effective solvent, with kinetics favoring direct intramolecular cyclization over side reactions.

## Supplementary Material



## Data Availability

The data underlying this study are available in the published article and its Supporting Information.

## Notes

Parameterized atomic charges for cation radical substrates as utilized in QM/MM simulations; oxidation potentials of substrates and products computed at the RI-MP2/6-31G*/CPCM­(methanol) and RI-MP2/cc-pVTZ/CPCM­(methanol) levels utilizing DFT-optimized geometries considered in Figure 2; cyclization reaction energies of cation radical intermediates **1a**, **7a**, **13a**, and **19a** as benchmarked by various DFT and MP2 levels of theory; and second oxidation potentials for protonated cyclic cation radical species **1b**, **7b**, **13b**, and **19b** (PDF)
