# Supplementary material for: Solvent Stabilization of Protic Oxonium/Ammonium Intermediates in Cation Radical Cyclization Reactions Investigated via Computational Approaches
Source: J Org Chem. 2026 Jul 7;91(28):9691–706. doi: 10.1021/acs.joc.6c00258 (PMC13386538; doi:10.1021/acs.joc.6c00258)
Supplement: Supplementary file 1 [file jo6c00258_si_001.pdf]

Supporting Information for "Solvent Stabilization of Protic Oxonium/Ammonium Intermediates in Cation Radical Cyclization Reactions Investigated via Computational Approaches"

Shahriar N. Khan,[†,‡] John H. Hymel,[†] and Jesse G. McDaniel[*,†]

†*School of Chemistry and Biochemistry, Georgia Institute of Technology, Atlanta, Georgia 30332, United States*

‡*Oak Ridge Leadership Computational Facility, Oak Ridge National Laboratory, Oak Ridge, Tennessee 37830, United States*

E-mail: mcdaniel@gatech.edu

Contents

S1 Parameterized atomic charges for the +1 oxidation states of the substrates **S2**

S2 Oxidation Potentials of Substrates and Products computed at RI-MP2/6-31G*/CPCM(methanol) and RI-MP2/cc-pVTZ/CPCM(methanol) level utilizing DFT Optimized Geometries **S4**

S3 Comparison of cyclization reaction energies for substrates 1a, 7a, 13a, and 19a computed at DFT/MP2 level of theory with various basis sets. **S4**

S4 Effect of Polarization Function on H Atom (6-31G) for the Hydrogen-bonded Complexes** **S5**

S5 Analysis of solvent exchange in QM/MM partition for cyclization reaction of cation radical substrate 1 **S6**

S6 Second oxidation potentials for protonated, cyclic cation radical species 1b, 7b, 13b, 19b. **S8**

S1 Parameterized atomic charges for the +1 oxidation states of the substrates

Table S1: Parameterized atomic charges computed at PBE0-D3/6-31G* level of theory utilizing distributed multi-pole analysis

**1a**		**7a**		**13a**		**19a**	
Atoms	Charge	Atoms	Charge	Atoms	Charge	Atoms	Charge
C	0.068	O	-0.727	C	0.111	C	-0.109
O	-0.360	C	0.284	O	-0.400	C	0.123
C	0.368	C	0.111	C	0.345	C	-0.227
C	0.019	C	0.018	C	-0.051	C	0.106
C	0.019	C	-0.023	C	0.056	C	-0.570
C	-0.136	C	-0.160	C	0.017	S	1.695
C	0.295	S	0.190	C	0.439	O	-0.753
O	-0.635	C	-0.080	N	-0.900	O	-0.738
H	0.164	C	0.079	S	1.786	N	-0.772
H	0.103	C	-0.061	O	-0.759	C	0.441
H	0.104	S	0.382	O	-0.792	C	0.056
H	0.033	H	0.489	C	-0.616	C	0.027
H	0.135	H	0.025	C	0.149	C	-0.061
H	0.077	H	-0.043	C	-0.235	C	-0.150
H	0.046	H	0.021	C	0.116	S	0.185
H	0.076	H	-0.020	C	-0.104	C	-0.068
H	0.059	H	0.068	C	-0.100	C	0.092
H	0.007	H	0.010	C	0.123	C	-0.053
H	0.070	H	0.105	H	0.145	S	0.251
H	0.488	H	0.011	H	0.082	C	0.094
		H	0.122	H	0.084	C	-0.107
		H	0.037	H	0.025	H	0.069
		H	0.005	H	0.134	H	0.068
		H	0.038	H	0.047	H	0.081
		H	0.117	H	0.015	H	0.157
				H	0.006	H	0.196
				H	0.015	H	0.359
				H	-0.010	H	-0.002
				H	-0.028	H	-0.060
				H	0.458	H	0.007
				H	0.184	H	-0.015
				H	0.156	H	0.004
				H	0.067	H	0.007
				H	0.067	H	0.114
				H	0.077	H	0.005
				H	0.102	H	0.111
				H	0.193	H	0.020
						H	-0.004
						H	0.015
						H	0.110
						H	0.195
						H	0.101

S2 Oxidation Potentials of Substrates and Products computed at RI-MP2/6-31G*/CPCM(methanol) and RI-MP2/cc-pVTZ/CPCM(methanol) level utilizing DFT Optimized Geometries

Table S2: Oxidation potentials $E^{\circ}_{rel,SHE}$ (V) of substrates and products computed at RI-MP2/6-31G*/CPCM(methanol) and in parenthesis RI-MP2/cc-pVTZ/CPCM(methanol) level on the DFT optimized geometries.

**Reactant**		**Product**		**Reactant**		**Product**	
Species Index	$E^{\circ}_{rel,SHE}$ (V)	Species Index	$E^{\circ}_{rel,SHE}$ (V)	Species Index	$E^{\circ}_{rel,SHE}$ (V)	Species Index	$E^{\circ}_{rel,SHE}$ (V)
**1**	1.25 (1.72)	**2**	1.81 (2.15)	**11**	1.06 (1.54)	**12**	0.65 (1.14)
**3**	1.02 (1.50)	**4**	1.52 (1.89)	**13**	1.28 (1.75)	**14**	2.75 (3.15)
**5**	0.86 (1.33)	**6**	1.60 (1.97)	**15**	0.90 (1.25)	**16**	1.20 (1.66)
**7**	0.92 (1.27)	**8**	1.43 (1.69)	**17**	0.91 (1.26)	**18**	0.96 (1.40)
**9**	1.32 (1.78)	**10**	0.93 (1.44)	**19**	0.94 (1.28)	**20**	2.32 (2.83)

S3 Comparison of cyclization reaction energies for substrates 1a, 7a, 13a, and 19a computed at DFT/MP2 level of theory with various basis sets.

Table S3: Reaction energies computed as $\Delta E = E_{\text{product}} - E_{\text{reactant}}$ in kJ/mol, where product is the cyclic structure (e.g. **1b**) and reactant is the uncyclized structure (e.g. **1a**). Calculations are done utilizing the CPCM (methanol) implicit solvent model with one explicit MeOH solvent.

Methods	**1a ↔ 1b**	**7a ↔ 7b**	**13a ↔ 13b**	**19a ↔ 19b**
**PBE0-D3/6-31G***	10.1	10.4	7.9	11.3
**PBE0-D3/6-31+G***	15.0	19.9	8.9	17.2
**PBE0-D3/6-311G***	12.4	15.3	11.4	17.7
**MP2/cc-pVTZ**	2.0	-	-14.2	5.7

S4 Effect of Polarization Function on H Atom (6-31G**) for the Hydrogen-bonded Complexes

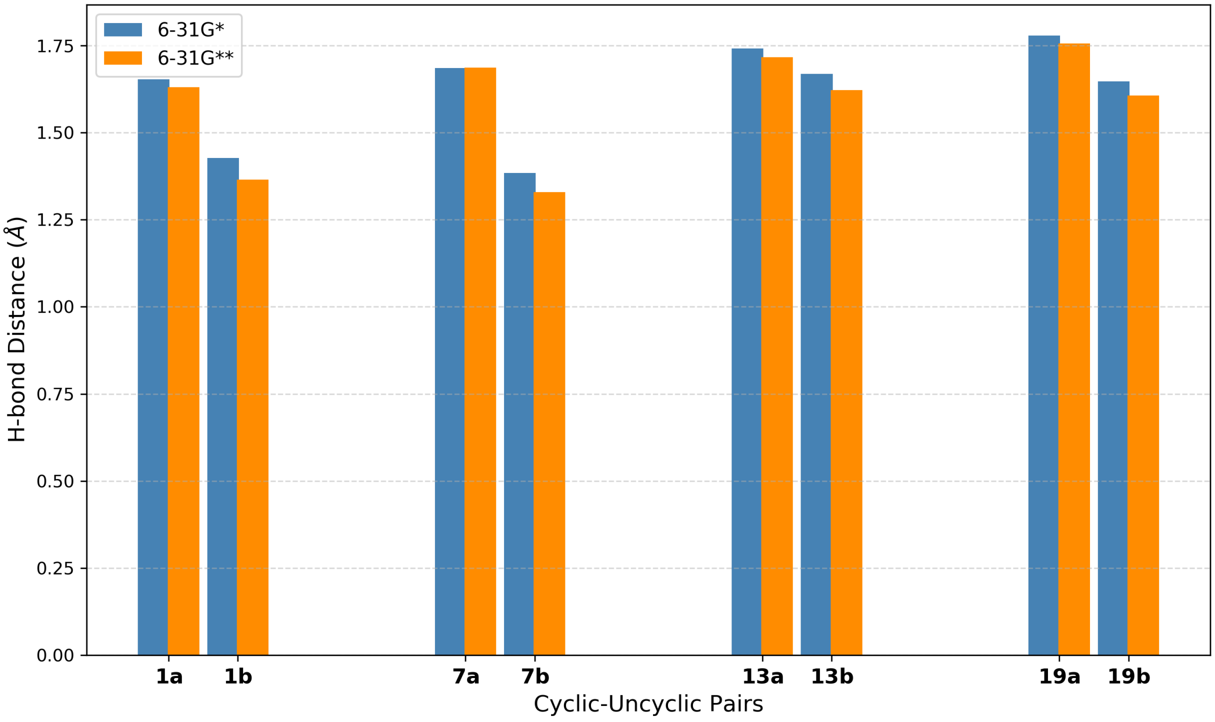

Figure S1: Comparison of the the hydrogen bond distances between protic H and the solvent (MeOH) for 6-31G* and 6-31G** basis sets.

In order to demonstrate the effect of basis set on predicted stability of the oxonium complex formation, we have compared the H-bond distances of the cyclic-uncyclic pairs of the alcohol and amine coupling species employing 6-31G* and 6-31G** basis sets (6-31G** has polarization function of H atom). All the cyclic products are stabilized by forming an oxonium complex with the solvent (MeOH) which is demonstrated by a short H-bond (~ 1.4 $\AA$ for **1b**, **7b** alcohol coupling and ~ 1.6 $\AA$ for **13b**, **19b** amine coupling cyclic products). Utilization of a polarization basis function on H atom (6-31G**) slightly shortens the H-bonds as compared to predicted geometries with the 6-31G* basis set. Differences are typically a few hundredths of an Angstrom or less, and are thus on par with errors/uncertainties introduced by e.g. choice of different exchange correlation functionals.

S5 Analysis of solvent exchange in QM/MM partition for cyclization reaction of cation radical substrate 1

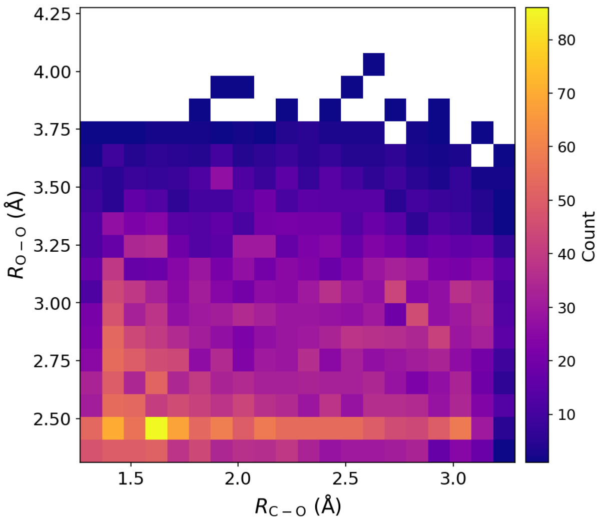

(a)

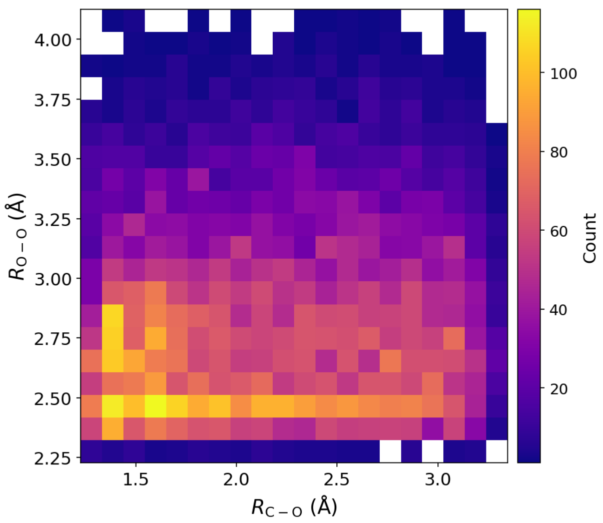

(b)

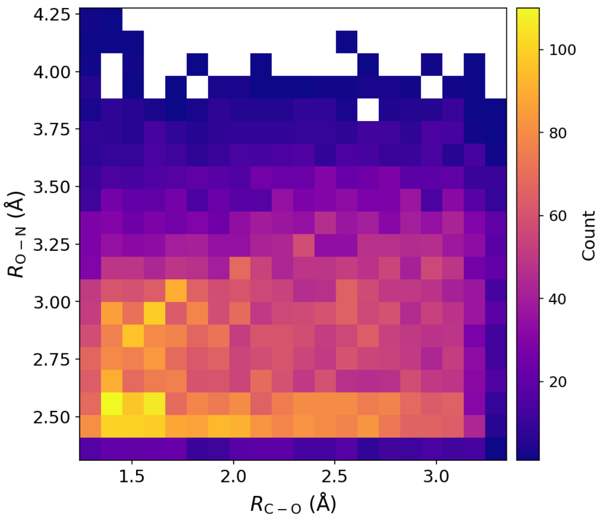

(c)

Figure S2: Analysis of QM solvent coordination distance to cation radical substrate **1** during cyclization reaction within solvent a) THF , b) MeOH, and c) ACN. The x-axis labeled R_{C-O} denotes the reaction coordinate, and y-axis labeled R_{O-O} (or R_{O-N}) denotes the distance between oxygen proton donor of substrate and basic oxygen (or nitrogen) atom of QM solvent molecule.

Within the QM/MM simulations discussed in the manuscript, one (and sometimes two) solvent molecules are added to the QM region that includes the cation radical substrate. The QM solvent molecule is chosen to be the closest solvent molecule in contact (e.g. h-bonding) with the protic alcohol or sulfonamide group of the substrate. This raises the question of whether during the course of the simulation the QM solvent molecule diffuses away from and "exchanges" with an MM solvent molecule, so that the latter is in close contact with the substrate. If such solvent exchange were to occur, it would separate the QM substrate and solvent in an undesired manner. In general, we find that the strong "complexing" interaction between QM solvent and substrate is sufficient to keep the QM solvent molecule localized near the QM substrate, without additional restraint. Figure S2 provides analysis of the QM solvent/substrate separation, for the example case of cation radical substrate **1** cyclization with one QM solvent molecule, as simulated with metadynamics (i.e. corresponding to reaction free energy simulations of Figure 9 in manuscript).

In Figure S2, the QM solvent/substrate distance is indicated by the y-axis, which plots the distance R_{O-O} (or R_{O-N}) between the oxygen atom of the substrate alcohol group and oxygen (or nitrogen atom) of QM solvent molecule. The x-axis indicates the reaction coordinate given by the cyclization bond formation distance R_{C-O} between alcohol oxygen and carbon cation radical center. Figure S2 a), b), and c) show histograms of roughly 10,000 simulation snapshots equally spaced over the entire metadynamics simulation for reactions in THF, MeOH, and ACN solvent respectively. As indicated by the histograms, it is most probable that the QM solvent molecule is located at separations $2.4 \leq R_{O-O} \leq 3.4$ Å from the substrate, implying that it is rare for the QM solvent molecule to "diffuse" more than $\sim$ 1 Å away from its close-contact "complexation" with the substrate. This indicates that the QM solvent molecule is well-localized in close proximity to the QM substrate, without the need for additional spatial restraints or more sophisticated adaptive QM/MM approaches. We note that because the histograms were constructed from metadynamics simulations, the probability distribution along the reaction coordinate is not reflective of the free energy

profile (since it includes added hills/bias), but this has no bearing on the analysis of solvent complexation distance.

S6 Second oxidation potentials for protonated, cyclic cation radical species 1b, 7b, 13b, 19b.

In the table below, we show computed second oxidation potentials for species **1b**, **7b**, **13b**, **19b**, which are protonated, cyclic cation radical structures. Energetics were computed at the PBE0/6-31G*/CPCM(methanol) level of theory. Computations were performed on cation radical species complexed with an explicit methanol solvent molecule, as solvent complexation is necessary to stabilize the protonated, cyclized cation radicals (Figure 5, manuscript). The "vertical" oxidation potential is that computed for the dication species at the same geometry as the optimized, cation radical species. The "adiabatic" oxidation potential is that computed for the dication species that is geometry optimized. The dications corresponding to substrates **1b** and **7b** undergo spontaneous deprotonation to the methanol solvent following oxidation (i.e. electrodissociation). Below the listed oxidation potentials, geometric bond lengths are given to indicate the extent of geometry relaxation upon second oxidation to the dication (and whether electrodissociation occurs). The "methanol H-O bondlength" is that between the acidic proton of the cation radical (or dication) substrate, and the oxygen acceptor atom of the complexed methanol molecule. Furthermore, we report the inner reorganization energy λ_{inner}, which is computed from dication and cation radical energetics utilizing the equilibration geometries of both species (equation below).

$$\lambda_{inner} = \frac{1}{2}\left((E_{+}(geom++) - E_{+}(geom+)) + (E_{++}(geom+) - E_{++}(geom++))\right) \quad (1)$$

where "E_{+}" indicates energy of cation radical, "E_{++}" indicates energy of dication,

"geom+" indicates geometry of optimized cation radical, "geom++" indicates geometry of optimized dication.

Table S4: Second oxidation potentials for protonated, cyclic cation radical species **1b**, **7b**, **13b**, **19b**, computed at PBE0/6-31G*/CPCM(methanol) level of theory with explicit methanol solvent molecular "complexed" to cation radical. Oxidation potentials are given w.r.t. SHE.

	**1b**	**7b**	**13b**	**19b**
vertical $E^{\circ}_{rel,SHE}$ (V)	0.77	0.34	0.65	0.41
adiabatic $E^{\circ}_{rel,SHE}$ (V)	-0.26	-0.47	-0.14	-0.18
$\lambda_{inner}(eV)$	0.93	0.63	0.91	0.47
**cation radical geometry**				
substrate H-O or H-N bondlength (Å)	1.06	1.08	1.07	1.07
methanol H-O bondlength (Å)	1.44	1.39	1.64	1.67
**dication geometry**				
substrate H-O or H-N bondlength (Å)	1.45	1.47	1.09	1.08
methanol H-O bondlength (Å)	1.05	1.05	1.55	1.61
